# Production of Potyvirus-Derived Nanoparticles Decorated with a Nanobody in Biofactory Plants

**DOI:** 10.3389/fbioe.2022.877363

**Published:** 2022-03-31

**Authors:** Maricarmen Martí, Fernando Merwaiss, Anamarija Butković, José-Antonio Daròs

**Affiliations:** Instituto de Biología Molecular y Celular de Plantas (Consejo Superior de Investigaciones Científicas-Universitat Politècnica de València), Valencia, Spain

**Keywords:** viral nanoparticle, VNP, plant virus, nanobody, potyvirus, zucchini yellow mosaic virus, tobacco etch virus

## Abstract

Viral nanoparticles (VNPs) have recently attracted attention for their use as building blocks for novel materials to support a range of functions of potential interest in nanotechnology and medicine. Viral capsids are ideal for presenting small epitopes by inserting them at an appropriate site on the selected coat protein (CP). VNPs presenting antibodies on their surfaces are considered highly promising tools for therapeutic and diagnostic purposes. Due to their size, nanobodies are an interesting alternative to classic antibodies for surface presentation. Nanobodies are the variable domains of heavy-chain (VHH) antibodies from animals belonging to the family Camelidae, which have several properties that make them attractive therapeutic molecules, such as their small size, simple structure, and high affinity and specificity. In this work, we have produced genetically encoded VNPs derived from two different potyviruses—the largest group of RNA viruses that infect plants—decorated with nanobodies. We have created a VNP derived from zucchini yellow mosaic virus (ZYMV) decorated with a nanobody against the green fluorescent protein (GFP) in zucchini (*Cucurbita pepo*) plants. As reported for other viruses, the expression of ZYMV-derived VNPs decorated with this nanobody was only made possible by including a picornavirus 2A splicing peptide between the fused proteins, which resulted in a mixed population of unmodified and decorated CPs. We have also produced tobacco etch virus (TEV)-derived VNPs in *Nicotiana benthamiana* plants decorated with the same nanobody against GFP. Strikingly, in this case, VNPs could be assembled by direct fusion of the nanobody to the viral CP with no 2A splicing involved, likely resulting in fully decorated VNPs. For both expression systems, correct assembly and purification of the recombinant VNPs was confirmed by transmission electron microscope; the functionality of the CP-fused nanobody was assessed by western blot and binding assays. In sum, here we report the production of genetically encoded plant-derived VNPs decorated with a nanobody. This system may be an attractive alternative for the sustainable production in plants of nanobody-containing nanomaterials for diagnostic and therapeutic purposes.

## Introduction

Nanotechnology is a rapidly expanding research area focused on the utilization of nanoscale particles for a broad range of applications. Numerous platforms have been developed to produce nanomaterials, ranging from chemical synthesis to repurposing bionanomaterials such as those derived from viral particles, known as viral nanoparticles (VNPs). These are virus-based formulations that can be used as building blocks for novel materials to support a range of functions of potential interest in medicine and nanotechnology, including vaccine platforms, targeted bioimaging and drug delivery (Steinmetz and Manchester, 2016; Steele et al., 2017; Chung et al., 2020; Rybicki, 2020). VNPs are receiving increasing attention due to their outstanding structural characteristics and easy functionalization (compared to synthetic nanoparticles). The advantages of VNPs include their ability to self-assemble with precise symmetry and polyvalency, their stability under a wide range of conditions, and their biocompatibility and biodegradability.

In particular, plant-derived VNPs provide unique nanoscale scaffolds for biotechnology applications in many areas (Marsian and Lomonossoff, 2016; Alemzadeh et al., 2018; Shukla et al., 2020a; Chung et al., 2021). Plant VNPs are attractive due to their desirable properties, including high yields and the rapid and scalable production that can be achieved in the laboratory or in molecular farming approaches, in which plants are used as VNP production factories (Lomonossoff and D’Aoust, 2016; Tusé et al., 2020). Furthermore, they present the advantage of being non-infectious in mammals—and thus inherently safe. Several plant viruses, both spherical and rod-shaped, have already been successfully deployed in the production of VNPs as scaffolds to support the display of peptides genetically encoded or chemically conjugated to structural viral proteins. Research in this area has focused on the development of plant viruses carrying antigenic epitopes from human or animal pathogens, with the aim of developing novel recombinant vaccines or diagnostic reagents (González-Gamboa et al., 2017; Chung et al., 2021; Peyret et al., 2021; Stander et al., 2021). The most widely used plant viruses for nanotechnology approaches are cowpea mosaic virus (CPMV) (Sainsbury et al., 2010; Beatty and Lewis, 2019; Ortega-Rivera et al., 2021), tobacco mosaic virus (TMV) (Röder et al., 2017; Lomonossoff and Wege, 2018), and potato virus X (PVX) (Lico et al., 2015; Le et al., 2019; Röder et al., 2019; Shukla et al., 2020b).

Viral genome engineering is the preferred strategy for modifying VNPs when the aim is to display small peptides. In particular, viral capsids are ideal for presenting epitopes by inserting them at an appropriate site on the selected viral coat protein (CP) (Cruz et al., 1996; Dickmeis et al., 2015; Röder et al., 2017). Although this CP-based decoration approach often results in the production of assembled particles, large cargoes may also produce reduced yield or defective particles. However, by incorporating wild-type subunits to produce mosaic particles, this problem can be alleviated (Cruz et al., 1996; Smolenska et al., 1998; Castells-Graells et al., 2018). This can be achieved by including the well-known picornavirus 2A splicing peptide between the fused proteins, utilizing the so-called overcoat strategy, which results in a mixed population of wild-type and modified proteins via a ribosomal co-translational skip mechanism.

**FIGURE 1:**
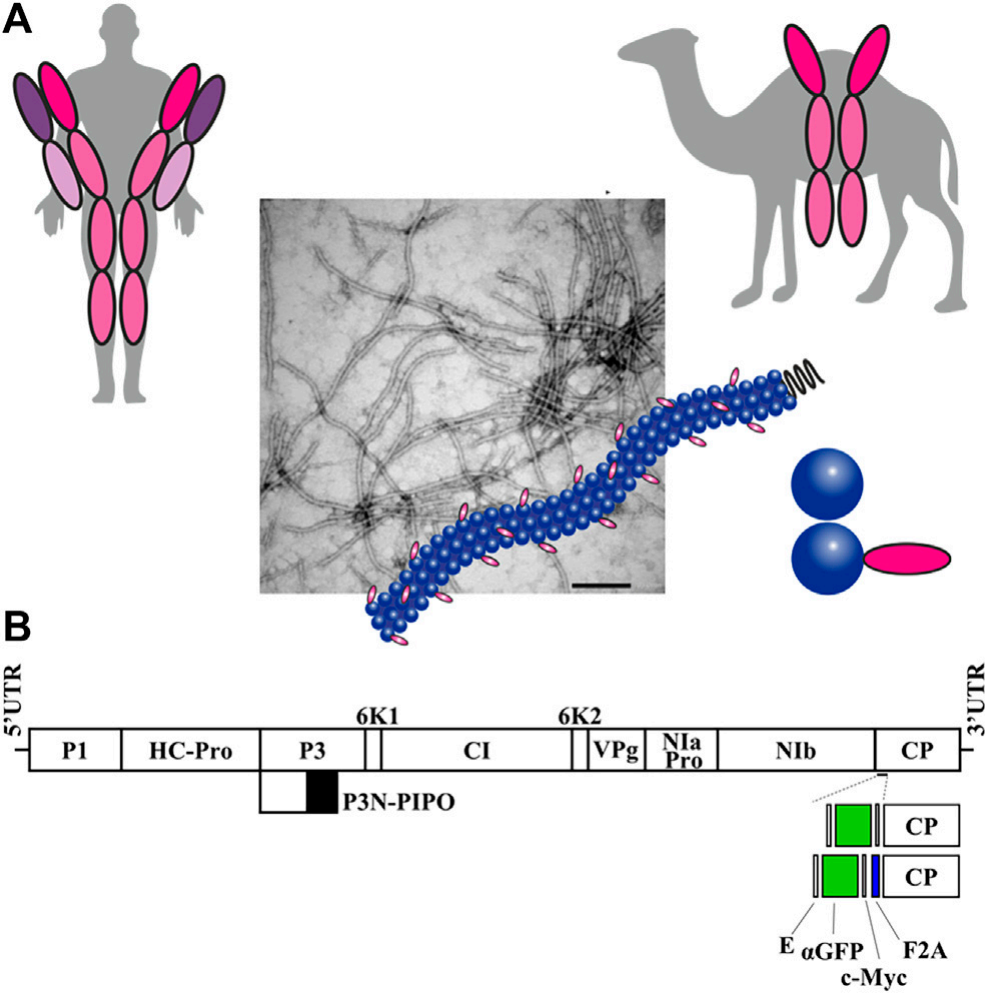
Production of potyvirus nanoparticles decorated with a nanobody in biofactory plants. **(A)** Schematic comparison of a conventional human antibody and a camelid heavy-chain antibody, from which single-domain antibodies or nanobodies are derived. A potyvirus virion partially decorated with nanobodies in its surface is also schematized. **(B)** Schematic representation of the ZYMV genome indicating the position where a heterologous sequence coding for an anti-GFP nanobody (αGFP) flanked with E and c-Myc epitopes was inserted, along with picornavirus F2A peptide. Lines represent ZYMV 5′ and 3′ UTRs; boxes represent P1, HC-Pro, P3, P3N-PIPO, 6K1, CI, 6K2, VPg, NIaPro, NIb, and CP cistrons, as indicated. Scale bar corresponds to 1000 nt.

Monoclonal antibodies (mAbs) represent a major class of biopharmaceutical products for therapeutic and diagnostic applications, with growing demand worldwide (Donini and Marusic, 2019; Chen, 2022). Plants have long been considered advantageous platforms for large-scale production of antibodies, because they constitute an inexpensive, efficient, and safe alternative system (Giritch et al., 2006; Juarez et al., 2016; Edgue et al., 2017). In addition to full-size mAbs, smaller antibody fragments capable of antigen binding are also actively studied and employed in medicine and research (Yusibov et al., 2016; Julve Parreño et al., 2018; Satheeshkumar, 2020; Malaquias et al., 2021; Wang et al., 2021). An interesting new alternative to mAbs are nanobodies (Muyldermans, 2013). These are the variable domain of heavy-chain (VHH) antibodies from animals belonging to the family Camelidae (Figure 1A), which have several properties that make them attractive therapeutic molecules. With molecular masses of 12–15 kDa, nanobodies are the smallest currently known antigen-binding proteins. Despite their small sizes, nanobodies bind their epitopes with high specificity and strong affinity (Muyldermans, 2013; Mitchell and Colwell, 2018; Wang et al., 2021).

Zucchini yellow mosaic virus (ZYMV) and tobacco etch virus (TEV) are two representative members of the genus *Potyvirus* within the family Potyviridae, the largest group of plant-infecting RNA viruses. They are flexuous rod-shaped viruses about 750 nm in length with a positive single-stranded RNA genome of approximately 10 kb (Revers and García, 2015). Several features make potyviruses appealing as expression vectors. Their expression via a polyprotein processed into a series of mature gene products facilitates production of heterologous proteins in an amount equimolar to the rest of the viral proteins. If the heterologous proteins are inserted flanked by the specific processing sites of a viral protease, they can be efficiently released from the polyprotein from a single vector (Kelloniemi et al., 2008; Bedoya et al., 2010; Cordero et al., 2018). The elongated nature of the virion allows for accommodating substantial amounts of foreign genetic material, as well as an extended surface for increased peptide exposure. Remarkably, some potyviruses have already been used as nanoscaffolds for short antigenic peptide presentation, yielding increased immunogenicity (Fernández-Fernández et al., 2002; Manuel-Cabrera et al., 2016; González-Gamboa et al., 2017; Yuste-Calvo et al., 2019). In addition, previous studies have shown that replacing the 33 amino-terminal amino acids of the ZYMV CP with a c-Myc tag does not affect the infectivity of the virus or its movement through the host plant (Arazi et al., 2001a).

We have previously reported the use of potyviral vectors for expressing heterologous proteins in plants, and even an entire biosynthetic pathway (Bedoya et al., 2010; Bedoya et al., 2012; Majer et al., 2015; Majer et al., 2017; Cordero et al., 2018; Martí et al., 2020; Houhou et al., 2022). In this study, we report the production of genetically encoded viral nanoparticles derived from ZYMV and TEV as nanoscaffolds for nanobody presentation. A picornavirus 2A peptide that mediates cleavage was used to modulate the degree of nanobody decoration on nanoparticles. More importantly, these recombinant virions carrying a nanobody against green fluorescent protein (GFP) were able to bind their antigen efficiently, demonstrating that these nanobodies were functional.

## Materials and Methods

### Plasmid Construction

Plasmid pGZYMV (Majer et al., 2017) contains the cDNA of an infectious wild-type (wt) variant of ZYMV (GenBank accession number KX499498), flanked by the cauliflower mosaic virus (CaMV) 35S promoter and terminator in a binary vector that derives from pCLEAN-G181 (Thole et al., 2007) (Figure 1B and Supplementary Figure S1). Derivatives from pGZYMV were constructed using standard molecular biology techniques, including polymerase chain reaction (PCR) amplification with the high-fidelity Phusion DNA polymerase (Thermo Scientific), DNA digestion with restriction enzymes followed by DNA ligation with T4 DNA ligase (Thermo Scientific), and Gibson assembly of DNA fragments (Gibson et al., 2009) using the NEBuilder HiFi DNA assembly master mix (New England Biolabs). pGZYMVΔ contains a ZYMV variant with a deletion from positions 8551 to 8640 of KX499498, corresponding to codons 4–33 of viral CP (Figure 1B and Supplementary Figure S1, ZYMVΔ). In pGZYMVΔ-αGFP, codons deleted from ZYMV CP were replaced by an anti-green fluorescent protein (αGFP) nanobody (Salema et al., 2013) flanked by E and c-Myc epitopes (Figure 1B and Supplementary Figure S1, ZYMVΔ-αGFP). In pGZYMVΔ-αGFP-F2A, a cDNA corresponding to foot-and-mouth disease virus 2A self-cleavage peptide (F2A) (Kim et al., 2011) was inserted between the αGFP and the deleted version of the CP (CPΔ) coding regions. The resulting viral recombinant clone was named ZYMVΔ-αGFP-F2A (Figure 1B and Supplementary Figure S1).

Plasmid pGTEVa (Bedoya et al., 2012) contains the cDNA of an infectious TEV variant with the GenBank accession number DQ986288 (G273A, A1119G), flanked by the CaMV 35S promoter and terminator in a binary vector derived from pCLEAN-G181. In pGTEV-αGFP, the αGFP nanobody cDNA, flanked by E and c-Myc epitopes, was inserted at the 5’ end of CP cistron. The three initial codons of TEV CP, including silent mutations, were duplicated to mediate NIaPro proteolytic processing. In pGTEV-αGFP-F2A, the cDNA corresponding to picornavirus F2A was inserted between the αGFP and the viral CP (Supplementary Figure S2).

Plasmid pEGFPSt contains the coding region of the enhanced GFP with a carboxy-terminal Twin-Strep tag (Schmidt et al., 2013) under the control of the bacteriophage T7 promoter and terminator for expression in *Escherichia coli* (Supplementary Figure S3).

### Plant Inoculation

Seeds of zucchini plants (*Cucurbita pepo* L. cv. MU-CU-16, accession BGV004370 from Centro de Conservación y Mejora de la Agrodiversidad Valenciana, Universitat Politècnica de València) were kept in darkness at 37°C for 2 days and moved to a growth chamber at 25°C with a 16/8 h day/night cycle to promote germination. Seedlings were sown into individual pots and maintained in a greenhouse at 25°C with a 16/8 h day/night cycle. The strain C58C1 of *Agrobacterium tumefaciens*, carrying the helper plasmid pCLEAN-S48 (Thole et al., 2007), was transformed with the plasmids containing the different ZYMV and TEV viral clones mentioned above. Transformed bacteria were selected in plates with 50 μg/ml rifampicin, 50 μg/ml kanamycin, and 7.5 μg/ml tetracycline. Individual colonies of the different clones were further grown for 24 h at 28°C in liquid media up to an optical density at 600 nm (OD_600_) of 0.5–1. Cells were harvested by centrifugation, resuspended in agroinoculation solution [10 mM MES-NaOH (pH 5.6), 10 mM MgCl_2_, and 150 μM acetosyringone] at OD_600_ of 0.5; the culture was further incubated for 2 h at 28°C. With a needleless syringe, cultures corresponding to ZYMV clones were used to infiltrate one cotyledon and one true leaf from 2-week old zucchini plants. After agroinoculation, plants were kept in a growth chamber at 25°C under a 12 h day/night photoperiod with an average photon flux density of 240 μmol m^−2^·s^−1^. Aliquots of symptomatic tissues from upper leaves and the equivalent tissues from mock-inoculated controls were harvested at 21 days post-inoculation (dpi), frozen in liquid nitrogen, and stored at −80°C until use.

*Nicotiana benthamiana* plants were grown at 25°C under a 16/8 h day/night cycle in growth chambers. Fully expanded upper leaves from plants 4–6 weeks old were used for agroinoculation, based on the protocol described above using *A. tumefaciens* cultures corresponding to the TEV clones. Immediately following infiltration, plants were watered and transferred to a growth chamber under a 12-h day/night and 25°C cycle. Aliquots of symptomatic tissues from upper leaves and the equivalent tissues from mock-inoculated controls were harvested at 14 dpi, frozen in liquid nitrogen, and stored at -80°C until use.

### RT-PCR Analysis of the Viral Progeny

Total RNA was purified from leaf tissue aliquots using silica-gel columns (Zymo Research) (Uranga et al., 2021a). For ZYMV analysis, aliquots of the RNA preparations were subjected to reverse transcription (RT) using the RevertAid reverse transcriptase (Thermo Scientific) and primer PI (5′-AGG​CTT​GCA​AAC​GGA​GTC​TAA-3′). Aliquots of the RT products were subjected to PCR amplification using the high-fidelity Phusion DNA polymerase and primers PII (5′-TGT​AAT​GCT​CCA​ATC​AGG​CAC​T-3′) and PIII (5′-CTG​CAT​TGT​ATT​CAC​ACC​TAG​T-3′), which are homologous and complementary, respectively, to sequences flanking the ZYMV CP cistron. For TEV analysis, reverse transcription was completed using primer PIV (5′-TCA​TAA​CCC​AAG​TTC​CGT​TC-3′), while PCR amplification was performed with primers PV (5′-CAT​CTG​TGC​ATC​AAT​GAT​CGA​A-3′) and PVI (5′-GTG​TGG​CTC​GAG​CAT​TTG​ACA​A-3′). PCR products were separated via electrophoresis in 1% (w/v) agarose gels that were subsequently stained with 1% (w/v) ethidium bromide.

### Western Blot Analysis

Aliquots of frozen tissue (approximately 50 mg) were ground with a mill (Star-Beater, VWR) using a 4-mm diameter steel ball for 1 min at 30 s^−1^. Three volumes of protein extraction buffer [60 mM Tris-HCl (pH 6.8), 2% (w/v) sodium dodecyl sulfate (SDS), 100 mM dithiothreitol (DTT), 10% (v/v) glycerol, 0.01% (w/v) bromophenol blue] were added. Samples were thoroughly vortexed, incubated for 5 min at 100°C, and clarified with centrifugation for 5 min. Aliquots of the supernatants were separated via SDS-polyacrylamide gel electrophoresis (PAGE) in 12.5% (w/v) polyacrylamide gels. Proteins were electro-blotted to polyvinylidene difluoride (PVDF) membranes for 1 h. Membranes were blocked in 5% (w/v) non-fat milk in washing buffer [10 mM Tris-HCl (pH 7.5), 154 mM NaCl, 0.1% (v/v) Nonidet P-40] for 1 h and were then incubated overnight at 4°C with various antibodies in blocking solution at 1:10,000 dilutions. Membranes were washed three times with washing buffer prior to detection. ZYMV CP and TEV CP were detected using polyclonal antibodies (Bioreba) conjugated to alkaline phosphatase (AP). c-Myc and E epitopes were detected using monoclonal antibodies (Thermo Scientific) conjugated to AP and horseradish peroxidase (HRP), respectively. AP and HRP were finally revealed using CSPD (Roche) and SuperSignal West Pico PLUS chemiluminescent (Thermo Scientific) substrates, respectively. Images were recorded using an Amersham ImageQuant 800 (Cytiva).

### Expression in *Escherichia coli* of Recombinant GFP and Purification

*E. coli* BL21(DE3)pLysS were electroporated with pEGFPSt, and transformed bacteria were selected in lysogenic broth (LB) plates containing 50 μg/ml ampicillin and 34 μg/ml chloramphenicol. A single colony was further grown at 37°C in 250 ml of LB liquid media containing the same antibiotics up to an OD_600_ of 0.6. Isopropyl β-d-1-thiogalactopyranoside (IPTG) was added to 0.4 mM, with culturing being continued for 3 h at the same temperature. Cells were pelleted at 7700 x g for 15 min, washed with water, pelleted again, and finally resuspended in 7.5 ml of water with a protease inhibitor cocktail (Complete; Roche). Bacteria were frozen and kept at −80°C until protein purification. The cell preparation was thawed and 1 ml of 1 M Tris-HCl (pH 8.0), 1 ml 10% (v/v) Nonidet-P40, 20 µL 0.5 M EDTA (pH 8.0), 200 µL 0.5 M DTT, 125 U benzonase (Millipore), and 10 mg lysozyme were added; this mix was incubated for 45 min at 4°C with gentle agitation. KCl (0.11 g) was then added, and the mix was further incubated for 15 min. Finally, 50 µL 1 M MgCl_2_ were added, and the preparation was brought to a final volume of 10 ml. This mix was then centrifuged at 84,500 x g at 4°C for 30 min; the supernatant was filtered using a 0.45 µm syringe filter.

Recombinant GFP with a carboxy-terminal TST was purified using affinity chromatography in native conditions with a 1 ml Strep-Tactin XT superflow column (IBA). Protein purification was conducted using an AKTA Prime Plus liquid chromatography system (GE Healthcare) at 4°C and a flow rate of 1 ml/min. The column was equilibrated with 10 ml of chromatography buffer [100 mM Tris-HCl (pH 8.0), 150 mM KCl, 1 mM EDTA, 5 mM MgCl_2_, 10 mM DTT, and 1% (v/v) Nonidet P40] before loading the protein extract. This column was then washed with 20 ml of chromatography buffer, and the recombinant GFP was finally eluted in 50 mM biotin in chromatography buffer with collection of 1-ml fractions. Purified protein fractions were analyzed via SDS-polyacrylamide gel electrophoresis [SDS-PAGE; 12.5% (w/v) polyacrylamide, 0.05% (w/v) SDS], followed by Coomassie blue staining. Bovine serum albumin standards were also run in the gel to quantify the GFP amount in each fraction.

### Virion Purification

Aliquots of symptomatic leaf tissue (0.5 g) were homogenized using a Polytron (Kinematica) in the presence of 1 ml of cold extraction buffer [0.5 M boric acid (pH 8.0), 1% (w/v) polyvinylpyrrolidone (PVP) 40, and 100 mM 2-mercaptoethanol]. Next, 0.25 ml chloroform and 0.25 ml CCl_4_ were added, and grinding continued. The mix was clarified via centrifugation for 15 min; the supernatant was recovered. While stirring the preparation on ice, we added 154 µL of a 40% (w/v) polyethylene glycol (PEG) 6000, 17.5% (w/v) NaCl solution. Stirring was maintained for 15 min. The mix was centrifuged for 5 min at 16,000 x g, and the supernatant was discarded. Sediment was resuspended by gentle agitation with a magnetic stir bar in 100 µL of 50 mM boric acid (pH 8.0), 5 mM EDTA, 0.25% (v/v) Triton X-100, and 25% (v/v) glycerol. Preparations were stored at −80°C until use.

### Electron Microscopy Analysis

Virion preparations were stained with 2% (w/v) phosphotungstic acid (PTA; pH 7.0), using the drop technique. The grid (carbon film coated only, 200 mesh, EMS) was layered on a 10 µL sample drop and incubated for 15 min. Grids were then washed with water, stained with 2% (w/v) PTA for 3 min, and dried at room temperature. Virion preparations were examined with a JEM-1400Flash (120 kV) electron microscope (JEOL).

## Results

### Plant-Based Production of ZYMV Nanoparticles Decorated with Anti-GFP Nanobodies

Previous work has shown that a ZYMV clone with a deletion corresponding to the 33 amino-terminal codons of the viral CP is infectious and that some heterologous sequences, notably c-Myc and 6xHis tags, can be fused to the amino-terminal end of this deleted version of CP without substantially impacting the infectivity and stability of the resulting recombinant clone (Arazi et al., 2001a). We wondered whether ZYMV would still support the expression of a substantially larger moiety, such as a nanobody, fused to the deleted version of CP to produce decorated nanoparticles. To examine this, we prepared a ZYMV recombinant clone in which CP codons from +4 to +33 (ZYMVΔ) were replaced by the cDNA of a nanobody specifically recognizing GFP (Salema et al., 2013) (ZYMVΔ-αGFP; Figure 1B and Supplementary Figure S1). αGFP nanobody was tagged with flanking c-Myc and E epitopes to facilitate detection (Figure 1B and Supplementary Figure S1). We maintained the first three codons of ZYMV CP to assure efficient NIaPro-mediated processing of the recombinant CP from the viral polyprotein (Adams et al., 2005). Since we foresaw potential limitations on the infectivity of a recombinant ZYMV fully decorated with the nanobody, we also prepared a derivative clone in which a picornavirus splicing domain—namely F2A (Kim et al., 2011)—was inserted between the nanobody and the CP to produce partially decorated viral nanoparticles (ZYMVΔ-αGFP-F2A; Figure 1B and Supplementary Figure S1).

**FIGURE 2:**
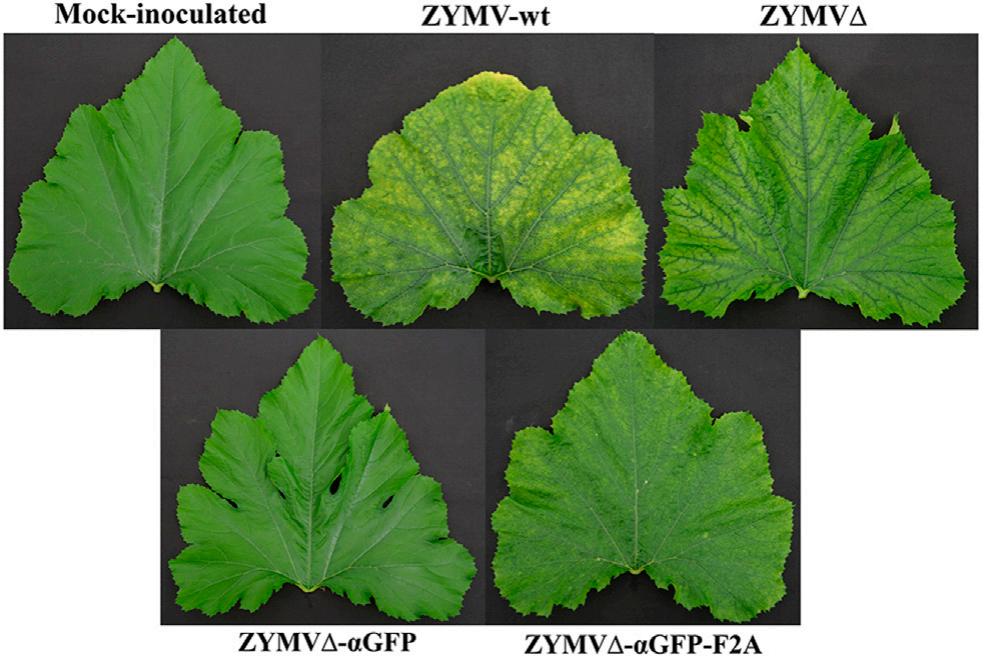
Pictures of representative upper leaves from zucchini plants mock-inoculated and agroinoculated with ZYMV-wt, ZYMVΔ, ZYMVΔ-αGFP, and ZYMVΔ-αGFP-F2A, as indicated, taken at 21 dpi.

**FIGURE 3:**
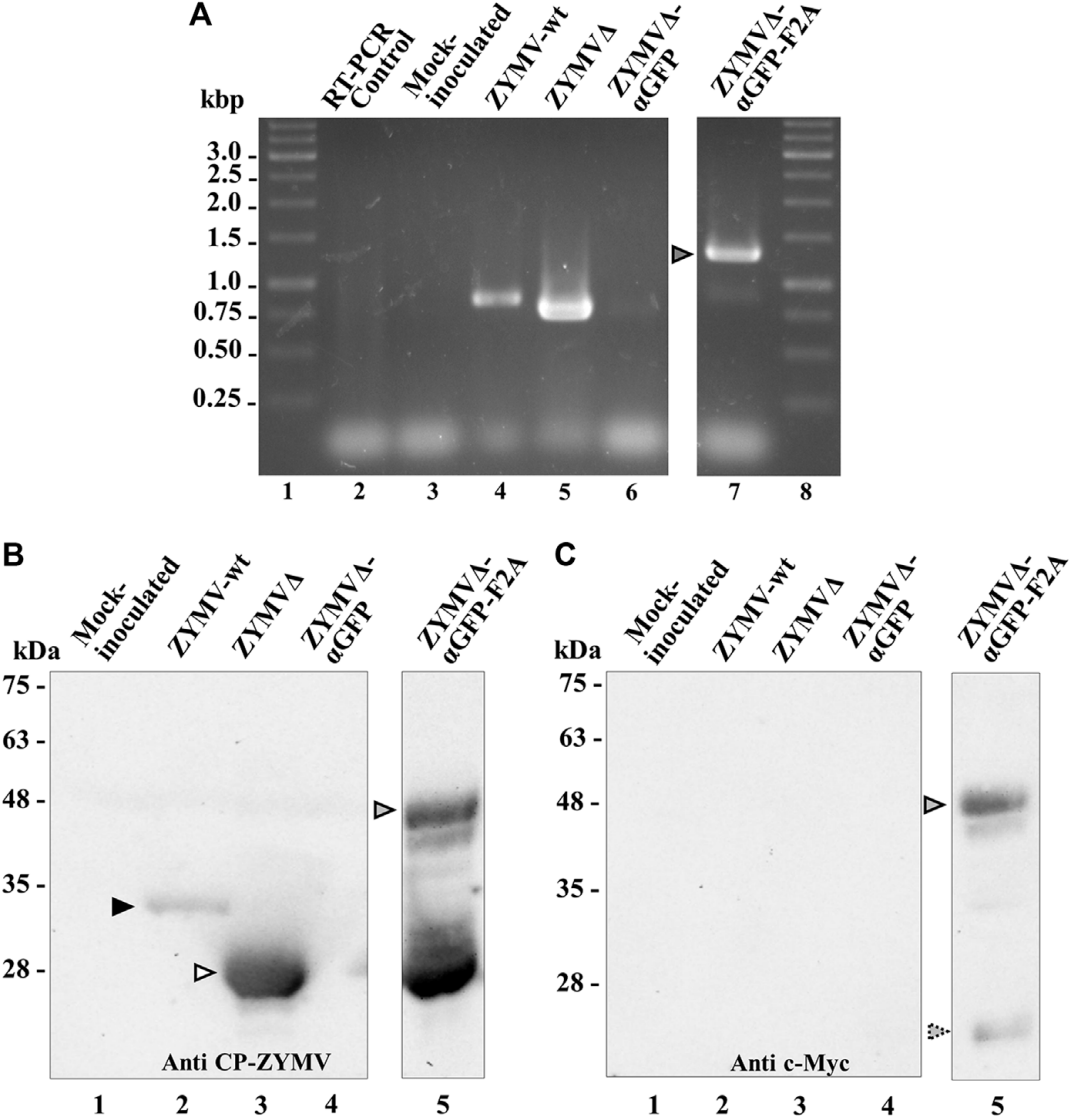
Analysis of zucchini tissues from plants inoculated with various ZYMV-derived recombinant clones at 21 dpi. **(A)** RT-PCR analysis of the progeny of recombinant ZYMV inoculated into zucchini plants. Representative samples from triplicate-inoculated plants are shown. Amplification products corresponding to the ZYMV CP region were separated via electrophoresis in an agarose gel, which was stained with ethidium bromide. Lanes 1 and 8, DNA marker ladder with sizes (in kbp) in the left; lane 2, RT-PCR control with no RNA added; lane 3, mock-inoculated plant; lanes 4 to 7, plants agroinoculated with ZYMV-wt, ZYMVΔ, ZYMVΔ-αGFP, and ZYMVΔ-αGFP-F2A, respectively. **(B and C)** Western blot analyses of protein extracts using an antibody against **(B)** ZYMV CP and **(C)** the c-Myc epitope fused to the αGFP nanobody. Proteins were separated by SDS-PAGE and transferred to a membrane. Lane 1, mock-inoculated plant; lanes 2 to 5, plants agroinoculated with ZYMV-wt, ZYMVΔ, ZYMVΔ-αGFP, and ZYMVΔ-αGFP-F2A, respectively. The positions and sizes of protein standards are indicated on the left. Black and white arrowheads indicate the positions of ZYMV CP and CPΔ, respectively. Gray and stripped arrowheads indicates the position of the αGFP-F2A-CPΔ fusion and free αGFP, respectively.

Zucchini plants were agroinoculated with ZYMV-wt and the various recombinant clones. Upper leaves of plants inoculated with ZYMV-wt showed typical symptoms of infection at 14 dpi. Similar symptoms, although milder, were observed in plants inoculated with ZYMVΔ. The symptoms in plants inoculated with ZYMVΔ-αGFP-F2A were particularly mild, while plants inoculated with ZYMVΔ-αGFP showed no apparent symptoms of infection (Figure 2). We then investigated viral ZYMV infection and the presence of the heterologous sequences corresponding to the αGFP nanobody in the viral progenies at 21 dpi, using RT-PCR amplification followed by electrophoretic analysis. RT-PCR products likely corresponding to the presence of full-length CP (850 bp) and the CPΔ (760 bp) were amplified from control plants inoculated with ZYMV-wt and ZYMVΔ, respectively (Figure 3A, lanes 4 and 5). Although they carried the nanobody sequence in the infectious clone, plants inoculated with ZYMVΔ-αGFP exhibited a slight band with the same position as that in ZYMVΔ (Figure 3A, lane 6), probably arising from a progeny that lost the exogenous sequence. Conversely, plants inoculated with ZYMVΔ-αGFP-F2A successfully produced a band whose position matched that expected for a recombinant clone maintaining the αGFP insert (1279 bp; Figure 3A, lane 7, gray arrowhead). Next, from equivalent tissue aliquots from upper leaves harvested at 21 dpi, the presence of ZYMV CP and the αGFP nanobody was assessed using western blot analysis with a polyclonal antibody against ZYMV CP and a monoclonal antibody against the c-Myc tag, respectively. Reaction with the anti-ZYMV CP antibody produced a single band in the lane corresponding to the plant inoculated with ZYMV-wt (Figure 3B, lane 2, black arrowhead). The position of this band, in comparison to those of protein size standards, suggests that it arose from ZYMV-wt CP. Lanes corresponding to plants inoculated with ZYMVΔ, and ZYMVΔ-αGFP-F2A showed bands with lower positions in the membrane, suggesting that they arose from the deleted version of ZYMV CP (Figure 3B, lanes 3 and 5, white arrowhead). Interestingly, a second intense band in the upper part of the membrane was also observed in the lane corresponding to a plant inoculated with ZYMVΔ-αGFP-F2A (Figure 3B, lane 5, gray arrowhead). A comparison with protein size standards suggests that it arose from a fusion between the deleted version of CP and the αGFP nanobody. No bands were observed in the lanes corresponding to the mock-inoculated plant and the plant inoculated with ZYMVΔ-αGFP (Figure 3B, lanes 1 and 4). Accordingly, reaction with the anti-c-Myc antibody exclusively produced bands in the lane corresponding to the plant inoculated with ZYMVΔ-αGFP-F2A (Figure 3C). The positions of the most intense bands in the membrane suggest detection of the αGFP-F2A-CPΔ fusion (Figure 3C, lane 5, gray arrowhead) and the free αGFP nanobody resulting from the F2A activity (Figure 3C, lane 5, striped arrowhead).

Together, these findings suggest that agroinoculation of the different ZYMV recombinant clones yielded infected plants. However, only ZYMVΔ-αGFP-F2A, which contains a picornavirus 2A self-cleavage domain—likely resulting in a partially nanobody-decorated viral nanoparticle—produced a substantial amount of αGFP-F2A-CPΔ fusion in infected plants.

### GFP-Binding Activity of Nanoparticles Derived from ZYMVΔ-F2A-αGFP

**FIGURE 4:**
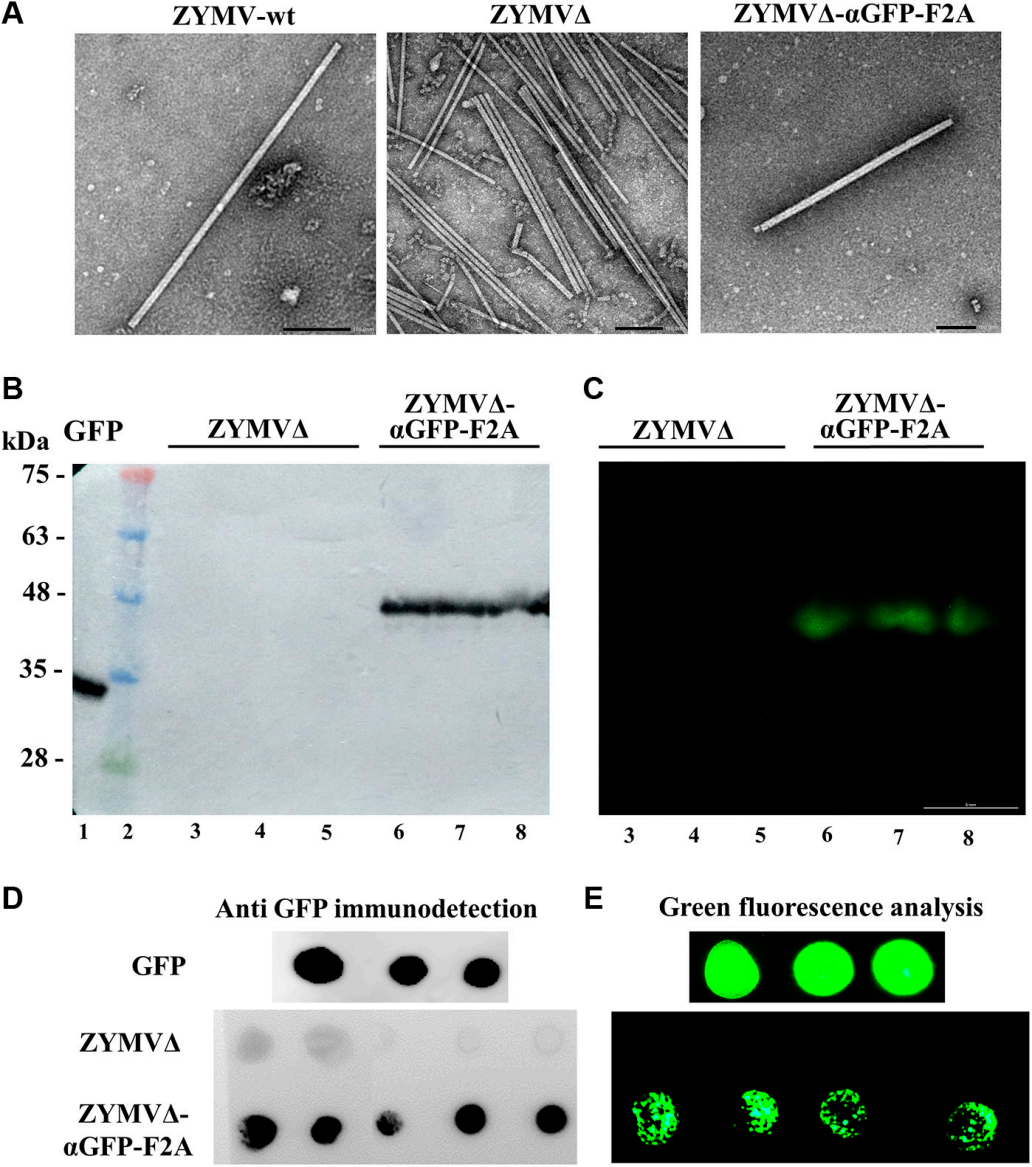
Antigen-binding capacity of ZYMV VNPs. **(A)** TEM micrographs of purified ZYMV-wt, ZYMVΔ, and ZYMVΔ-αGFP-F2A virions, as indicated. Scale bar is 100 nm. **(B**, **C)** GFP-binding properties of ZYMV αGFP-F2A-CPΔ capsomers. Virion preparations from zucchini plants infected with ZYMVΔ and ZYMVΔ-αGFP-F2A were separated by SDS-PAGE in triplicate, and the proteins were transferred to a membrane. The membranes were incubated with recombinant GFP and washed; GFP was revealed by **(B)** reaction with a specific antibody or **(C)** by directly imaging the green fluorescence. Lane 1, recombinant GFP; lane 2, protein standards with sizes in kDa on the left; lanes 3 to 8, virion preparations from plants infected with ZYMVΔ (lanes 3–5) or ZYMVΔ-αGFP-F2A (lanes 6–8). **(D**, **E)** Dot-blot analysis of the GFP-binding activity of ZYMVΔ-αGFP-F2A virions. Aliquots of virion preparations from plants infected with ZYMVΔ and ZYMVΔ-αGFP-F2A, as indicated, were spotted on membranes that were incubated with recombinant GFP, and GFP detected with a specific antibody **(D)** or the fluorescence directly analyzed using a stereomicroscope **(E)**. Membranes in which aliquots of recombinant GFP were spotted were used as a positive control.

Virions were next purified from zucchini plants agroinoculated with ZYMV-wt, ZYMVΔ, and ZYMVΔ-αGFP-F2A at 21 dpi. Transmission electron microscope (TEM) analysis of virion preparations showed the presence in all cases of the elongated viral nanoparticles, with a length of approximately 750 nm expected for ZYMV (Figure 4A). Next, we separated the virion preparations by SDS-PAGE and transferred the proteins to PVDF membranes that were incubated with recombinant GFP. After washing the membranes, binding of GFP was detected by using an anti-GFP antibody or by directly analyzing the green fluorescence. When an anti-GFP antibody was used to detect the presence of GFP (Figure 4B), bands were observed in a position of the membrane corresponding to the expected migration of the αGFP-F2A-CPΔ fusion capsomers. When analyzing the membrane with a fluorescence stereomicroscope, intense green fluorescent signals at exactly the same position were observed (Figure 4C). We observed no such reaction bands or fluorescent signals in the lanes where ZYMVΔ virions were separated. Finally, aliquots of the virion preparations were directly spotted onto PVDF membranes that were also incubated with recombinant GFP. After washing the membranes, binding of GFP was detected again—either indirectly by reaction with an anti-GFP antibody or directly using a fluorescence stereomicroscope. In contrast to spots containing nanoparticles purified from plants inoculated with ZYMVΔ, those corresponding to ZYMVΔ-αGFP-F2A showed strong reaction with the anti-GFP antibody (Figure 4D), along with intense green fluorescence (Figure 4E). Overall, these results strongly suggest that zucchini plants infected with recombinant ZYMVΔ-αGFP-F2A accumulate viral nanoparticles partially decorated with an αGFP nanobody, due to the conditional activity of picornaviral F2A peptide, and that these ZYMV-derived nanoparticles exhibit specific GFP-binding activity.

### Plant-Based Production of TEV Nanoparticles Decorated with Anti-GFP Nanobodies

**FIGURE 5:**
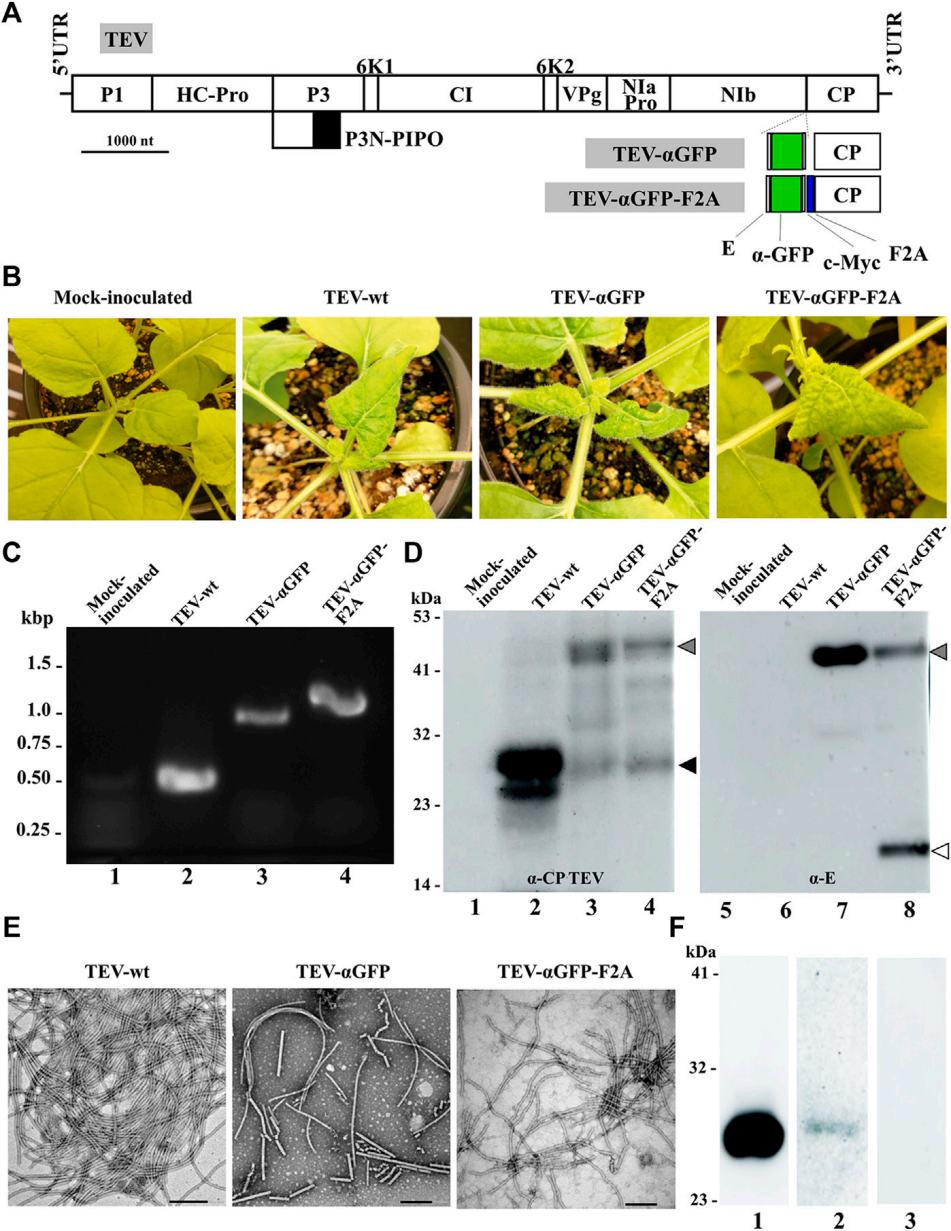
Production of TEV-derived nanoparticles decorated with a nanobody in biofactory plants. **(A)** Schematic representation of the TEV genome indicating the position where a heterologous sequence coding for an αGFP flanked with E and c-Myc epitopes was inserted with or without the picornavirus F2A peptide. Lines represent the TEV 5′ and 3′ UTRs; boxes represent the P1, HC-Pro, P3, P3N-PIPO, 6K1, CI, 6K2, VPg, NIaPro, NIb, and CP cistrons, as indicated. Scale bar corresponds to 1000 nt. **(B)** Pictures of representative upper leaves from *N. benthamiana* plants agroinoculated with TEV-wt, TEV-αGFP, and TEV-αGFP-F2A, as indicated, taken at 14 dpi. **(C)** RT-PCR analysis of the progeny of recombinant TEV inoculated into *N. benthamiana* plants. Representative samples from triplicate-inoculated plants are shown. Amplification products around the TEV CP region were separated by electrophoresis in an agarose gel, which was stained with ethidium bromide. DNA marker ladder with sizes (in kbp) on the left; lane 1, mock-inoculated plant; lanes 2 to 4, plants agroinoculated with TEV-wt, TEV-αGFP, and TEV-αGFP-F2A, respectively. **(D)** Western blot analyses of protein extracts using antibodies against TEV CP (left panel) and the E epitope fused to the αGFP nanobody (right panel). Proteins were separated by SDS-PAGE and transferred to a membrane. Lane 1 and 5, mock-inoculated plant; lanes 2 and 6, plant agroinoculated with TEV-wt; lanes 3 and 7, plant agroinoculated with TEV-αGFP; lanes 4 and 8, plant agroinoculated with TEV-αGFP-F2A. The positions and sizes of protein standards are indicated on the left. Arrowheads indicates the positions of TEV CP (black), free αGFP (white), and the αGFP-F2A-CP fusion (gray). **(E)** TEM micrographs of purified TEV-wt, TEV-αGFP, and TEV-αGFP-F2A virions, as indicated. Scale bar indicates 200 nm. **(F)** Assays to evaluate the antigen-binding capacity of the TEV-derived VNP, with nanobodies against GFP by western blot. GFP was separated by SDS-PAGE by triplicate, and the proteins were transferred to a membrane. The first lane was incubated with a commercial anti GFP antibody conjugated to HRP; the second lane was incubated with virion preparations from plants infected with TEV-αGFP-F2A and a secondary anti E-HRP; the third lane was incubated only with the anti E-HRP antibody.

We next explored whether the same concept could be extended to TEV, another potyvirus frequently used for expressing heterologous proteins in plants of *N. benthamiana*, the preferred production platform in molecular farming (Peyret and Lomonossoff, 2015; Bally et al., 2018; Goulet et al., 2019). To this end, we cloned a cDNA coding for the αGFP nanobody fused to the 5’ end of the viral CP cistron (Figure 5A). In this case, unlike with ZYMV, we inserted the exogenous sequence without deleting any subsequent region of the CP coding sequence. In addition to the viral clone with the directly fused nanobody, we also built a derived viral clone including the self-cleaving F2A domain, based on the results obtained with ZYMV. *N. benthamiana* plants were agroinoculated with TEV-wt and the different recombinant clones (TEV-αGFP and TEV-αGFP-F2A). Upper leaves of plants inoculated with TEV-wt showed typical symptoms of infection at 7 dpi, while plants inoculated with TEV-αGFP-F2A showed similar symptoms just 2 days later. Strikingly, unlike the results with ZYMV, the TEV-αGFP clone carrying the αGFP directly fused to the CP developed infection symptoms in the upper leaves at 11 dpi (Figure 5B). To analyze the outcome of the infection for each viral clone, we collected tissue from infected upper leaves at 14 dpi and evaluated the presence of the heterologous sequence corresponding to the αGFP nanobody in the viral progenies, using RT-PCR amplification followed by electrophoretic analysis. As expected, a 500-bp RT-PCR product was amplified from plants inoculated with TEV-wt (Figure 5C, lane 2). Accordingly, plants inoculated with TEV-αGFP and TEV-αGFP-F2A produced bands whose positions matched those expected for recombinant clones containing the αGFP fused sequence, without or with F2A, respectively (953 and 1028 bp; Figure 5C, lanes 3 and 4). We next analyzed the accumulation of the CP and the fused αGFP by western blot assays, using a polyclonal antibody against TEV CP and a monoclonal antibody against the E tag, respectively. Reaction with the anti-TEV CP antibody produced a single band corresponding to the expected size (30 kDa), which was observed in the lane corresponding to the plant inoculated with TEV-wt (Figure 5D, lane 2, black arrowhead), while the plant inoculated with TEV-αGFP-F2A showed two bands with similar intensity whose sizes corresponded to the fused αGFP-F2A-CP (48 kDa) and the free CP (Figure 5D, lane 4, gray and black arrowheads, respectively). Interestingly, a similar two-band pattern was observed for the plant inoculated with TEV-αGFP, although the intensity of the fused-CP band (46 kDa) was stronger (Figure 5D, lane 3). This result, although striking, could reflect the presence of a small subpopulation of viral progeny that lost the inserted sequence or the partial *in vivo* proteolytic cleavage of the inserted polypeptide extension. Reaction with the anti-E antibody showed the presence of a band arising from a protein of the expected size for the αGFP and CPΔ fusion in the lanes corresponding to plants inoculated with TEV-αGFP and TEV-αGFP-F2A (Figure 5D, lanes 7 and 8, gray arrowhead). As expected, free αGFP nanobody was detected only in the last lane, as a result of the activity of the F2A peptide (Figure 5D, lane 8, white arrowhead).

Next, VNPs were purified from *N. benthamiana* plants agroinoculated with TEV-wt, TEV-αGFP, and TEV-αGFP-F2A at 14 dpi. TEV-wt VNPs were obtained with an estimated yield of 50 mg per kg of fresh infected tissue; recombinant VNPs yield decreased to around 5 and 10 mg per kg for TEV-αGFP and TEV-αGFP-F2A, respectively. TEM analysis of virion preparations shows the presence in all cases of the elongated and flexuous viral nanoparticles, with a length of approximately 750 nm expected for TEV (Figure 5E). To finally analyze the functionality of the nanobodies, we ran an aliquot of purified recombinant GFP by SDS-PAGE and performed a western blot assay using the VNPs derived from TEV-αGFP-F2A as a primary antibody. The presence of the VNPs interacting with GFP in the transferred membrane was revealed using an anti-E antibody conjugated to HRP (Figure 5F, lane 2). As a positive control, we detected the presence of GFP with a commercial anti-GFP antibody conjugated to HRP (Figure 5F, lane 1), while the negative control lane was incubated only with anti-E-HRP (Figure 5F, lane 3). We successfully detected GFP using our αGFP-decorated VNP derived from TEV, showing that the CP-fused nanobodies are functional after virion assembly. These results indicate that agroinoculation of TEV recombinant clones coding for a CP-fused αGFP nanobody resulted in infections and the efficient production of VNPs carrying functional recombinant nanobodies.

## Discussion

Plant viruses-derived VNPs have been engineered to be used as vaccines or diagnostic reagents (Lico et al., 2015; Rybicki, 2020; Chung et al., 2021) as well to be used as carriers for drug delivery, targeted bioimaging and cancer immunotherapies (Shukla and Steinmetz, 2016; Chung et al., 2020). In these applications, virion shape plays a key role. For instance, elongated virions better accommodate large amounts of foreign genetic material; due to their higher aspect ratio, an elongated shape may present ligands more effectively than spherical counterparts. Furthermore, in clinical applications, rod filamentous viruses have better passive tumor homing, deeper tissue penetration, and more possibilities to resist immune detection and macrophage uptake than spherical counterparts (Bruckman et al., 2014; Shukla et al., 2015; Shukla et al., 2020a). Strikingly, limited attention has been paid to potyviruses, the largest group of plant filamentous viruses. Nevertheless, viruses of this type are amenable to nanotechnology, as shown by recent reports on their genetic modification to expose epitopes on their surface, suggesting than these viruses may also have potential biomedical applications (Sánchez et al., 2013; Yuste-Calvo et al., 2019; Frías-Sánchez et al., 2021).

Nanobodies have arisen in several fields, garnering outstanding interest as targeting molecules for bioimaging probes in cancer treatment and research (Oliveira et al., 2013), as therapeutics and diagnostic reagents against human diseases and pathogens (De Meyer et al., 2014; Wang et al., 2016; Wang et al., 2020), or in agriculture to mitigate the adverse effects of pesticide use (Ghannam et al., 2015; De Coninck et al., 2017; Hemmer et al., 2018). Nevertheless, nanobodies face several constraints: due to their small sizes, they are quickly eliminated from the bloodstream, and in some circumstances, their benefits must be enhanced by combined administration with other treatments (Salvador et al., 2019). The aim of this study was to produce genetically encoded VNPs derived from potyviruses as an alternative platform for nanobody display in plant biofactories. In this regard, the use of spherical VLPs for nanobody display has been reported by means of assembling recombinant fused capsids (Peyret et al., 2015). In contrast, in this study, we have functionalized the CP subunits of ZYMV and TEV to obtain flexuous rod-shaped biomaterials for nanobody presentation via genetic fusion. A nanobody against GFP was chosen as a proof of concept.

ZYMV is an important pathogen of cucurbits that reduces yields in some of the most important crops worldwide, such as squash, melon or cucumbers (Gal-On, 2007; Simmons et al., 2013). So far, ZYMV has been used in biotechnology to produce antiviral and antitumor proteins or metabolites (Arazi et al., 2001b; Arazi et al., 2002; Cordero et al., 2017; Majer et al., 2017), or to fortify zucchini fruits with carotenoids (Houhou et al., 2022). Furthermore, zucchini plants may be suitable biofactories due to their rapid growth rate and capacity for high biomass production over a short period of time, thereby facilitating production of a high amount of the desired product. By introducing selected epitopes at an appropriate position on the viral CP, VNPs are ideal scaffolds for their presentation. It has been reported that the ZYMV CP amino-terminal end is not necessary for infection and can even be partially replaced by a non-viral sequence (Arazi et al., 2001a). We decided to generate this shorter CP harboring a nanobody for its presentation. Potyvirus CP deletions and insertion of heterologous sequences at the carboxy-terminal end are unlikely to be viable due to the presence of RNA elements required for genome amplification in this region of the genome (Haldeman-Cahill et al., 1998). First, we confirmed that having a truncated CP with thirty fewer amino acids from its amino terminal end was not an impediment for infection (Figure 2 and Figure 3). Conversely, the direct fusion of the nanobody to CP did not result in plants with infection symptoms. Although several peptides have been successfully presented in this way in other systems, the size of peptide fusions remains a limiting factor that can impair virus assembly. Thus far, the maximum epitope sequences that have been displayed as direct CP fusions on the particle surface have been 60 and 113 amino acid residues in length, respectively (Uhde-Holzem et al., 2016; Röder et al., 2018). In contrast, our αGFP nanobody flanked by the E and c-Myc tags consisted of 148 amino acids. In order to display larger sequences, the 2A splicing peptide from picornaviruses can be inserted between the polypeptide of interest and the CP (Uhde-Holzem et al., 2010; Kim et al., 2011; Dickmeis et al., 2015). Different picornaviral 2A peptides showed different cleavage efficiency *in vivo* in different cell lines and organisms (Kim et al., 2011). In this way, by using different 2A peptides, the degree of decoration of the viral nanoparticles can be modulated, thereby increasing the chances of obtaining viable viruses that accumulate at optimal concentrations. Among the different picornavirus 2A peptides, F2A exhibits less efficient *in vivo* cleavage (Kim et al., 2011); therefore a higher degree of VNP decoration is expected. Despite the expected outcome of not obtaining satisfactory results with the direct fusion of the nanobody to the CP, the addition of a 2A peptide allowed us to obtain partially decorated ZYMV VNPs. After purification, the morphology of ZYMVΔ-αGFP-F2A VNPs was assessed via TEM, and no differences were found between these and the ZYMVΔ or ZYMV-wt VNPs (Figure 4A). Next, we evaluated the binding efficacy of the ZYMVΔ-αGFP-F2A VNP to its antigen. Interestingly, we proved that these VNPs can bind GFP successfully (Figure 4), suggesting that potyvirus-derived nanoparticles were decorated with functional multivalent nanobodies.

Next, TEV was tested as a source of scaffolds for nanobody presentation. This potyvirus was chosen because it has traditionally been used as a model for RNA viruses research (Bedoya et al., 2012) and because we have extensively exploited it as a biotechnological tool previously (Bedoya and Daròs, 2010; Majer et al., 2017; Martí et al., 2020; Uranga et al., 2021b). Furthermore, in contrast to most of the ZYMV strains (Gal-On, 2007; Cordero et al., 2017), TEV much more effectively infects *N. benthamiana*, which can be grown at high density in a matter of weeks and is the workhorse plant for excellence in plant molecular farming (Peyret and Lomonossoff, 2015). Interestingly, in this case, assembly was not compromised by the direct fusion of the nanobody to the TEV CP (Figure 5). Despite TEV-αGFP not carrying the F2A peptide, a slight band with a size corresponding to the free CP was also detected by western blot in some samples (Figure 5D). On the one hand, this could reflect the presence of a small subpopulation in the viral progeny that lost the inserted sequence. On the other hand, free CP could also result from *in vivo* cleavage of some of the protruding nanobodies, as previously proposed for PVX-derived VNPs (Röder et al., 2018). To shed some light on this, future work on time-course analysis of viral vector progeny would inform about insert stability and would help to optimize harvest time. All in all, although it may facilitate virus amplification and probably increase the viral load in the plant, the inclusion of the F2A peptide does not seem to be a necessary requirement for assembly of TEV-derived VNPs decorated with nanobodies.

The results presented in this study should allow us to extend and develop a set of plant VNPs for nanobody presentation. In order to avoid severe off-target effects of systemic drugs in medicine, several efforts are being made to develop targeted therapy using VNPs, in which drugs are either delivered specifically to tumor cells or activated specifically within them (Shukla et al., 2020a; Chung et al., 2020; Thuenemann et al., 2021). Therefore, further attempts in this area will involve the presentation of nanobodies with medicinal value. In addition, nanobodies presented on multivalent nanoparticles could improve the diagnosis and treatment of diseases. In summary, we report on genetically encoded potyvirus-derived VNPs used as scaffolds for nanobody presentation. This study sets the framework for the development of new tools derived from plant VNPs as nanomaterials useful in medicine or in other fields, according to the nanobodies of interest used.

## Data Availability

The original contributions presented in the study are included in the article/Supplementary Material, further inquiries can be directed to the corresponding author.

## References

[B1] Adams M. J. Antoniw J. F. Beaudoin F. (2005). Overview and Analysis of the Polyprotein Cleavage Sites in the Family Potyviridae. Mol. Plant Pathol. 6, 471–487. Available at: isi:000230765100009. 10.1111/j.1364-3703.2005.00296.x 20565672

[B2] Alemzadeh E. Dehshahri A. Izadpanah K. Ahmadi F. (2018). Plant Virus Nanoparticles: Novel and Robust Nanocarriers for Drug Delivery and Imaging. Colloids Surf. B: Biointerfaces 167, 20–27. 10.1016/j.colsurfb.2018.03.026 29625419

[B3] Arazi T. Lee Huang P. Huang P. L. Zhang L. Moshe Shiboleth Y. Gal-On A. (2002). Production of Antiviral and Antitumor Proteins MAP30 and GAP31 in Cucurbits Using the Plant Virus Vector ZYMV-AGII. Biochem. Biophysical Res. Commun. 292, 441–448. 10.1006/bbrc.2002.6653 11906182

[B4] Arazi T. Shiboleth Y. M. Gal-On A. (2001a). A Nonviral Peptide Can Replace the Entire N Terminus of Zucchini Yellow Mosaic Potyvirus Coat Protein and Permits Viral Systemic Infection. J. Virol. 75, 6329–6336. 10.1128/JVI.75.14.6329-6336.2001 11413299 PMC114355

[B5] Arazi T. Slutsky S. G. Shiboleth Y. M. Wang Y. Rubinstein M. Barak S. (2001b). Engineering Zucchini Yellow Mosaic Potyvirus as a Non-pathogenic Vector for Expression of Heterologous Proteins in Cucurbits. J. Biotechnol. 87, 67–82. 10.1016/S0168-1656(01)00229-2 11267700

[B6] Bally J. Jung H. Mortimer C. Naim F. Philips J. G. Hellens R. (2018). The Rise and Rise of Nicotiana Benthamiana: A Plant for All Reasons. Annu. Rev. Phytopathol. 56, 405–426. 10.1146/annurev-phyto-080417-050141 30149789

[B7] Beatty P. H. Lewis J. D. (2019). Cowpea Mosaic Virus Nanoparticles for Cancer Imaging and Therapy. Adv. Drug Deliv. Rev. 145, 130–144. 10.1016/j.addr.2019.04.005 31004625

[B8] Bedoya L. C. Daròs J.-A. (2010). Stability of Tobacco Etch Virus Infectious Clones in Plasmid Vectors. Virus. Res. 149, 234–240. 10.1016/j.virusres.2010.02.004 20152868

[B9] Bedoya L. C. Martínez F. Orzáez D. Daròs J.-A. (2012). Visual Tracking of Plant Virus Infection and Movement Using a Reporter MYB Transcription Factor that Activates Anthocyanin Biosynthesis. Plant Physiol. 158, 1130–1138. 10.1104/pp.111.192922 22238422 PMC3291247

[B10] Bedoya L. Martínez F. Rubio L. Daròs J.-A. (2010). Simultaneous Equimolar Expression of Multiple Proteins in Plants from a Disarmed Potyvirus Vector. J. Biotechnol. 150, 268–275. 10.1016/j.jbiotec.2010.08.006 20728479

[B11] Bruckman M. A. Randolph L. N. VanMeter A. Hern S. Shoffstall A. J. Taurog R. E. (2014). Biodistribution, Pharmacokinetics, and Blood Compatibility of Native and PEGylated Tobacco Mosaic Virus Nano-Rods and -spheres in Mice. Virology 449, 163–173. 10.1016/j.virol.2013.10.035 24418549 PMC3906584

[B12] Castells-Graells R. Lomonossoff G. P. Saunders K. (2018). Production of Mosaic Turnip Crinkle Virus-like Particles Derived by Coinfiltration of Wild-type and Modified Forms of Virus Coat Protein in Plants. Methods Mol. Biol. 1776, 3–17. 10.1007/978-1-4939-7808-3_1 29869231

[B13] Chen Q. (2022). Development of Plant-Made Monoclonal Antibodies against Viral Infections. Curr. Opin. Virol. 52, 148–160. 10.1016/j.coviro.2021.12.005 34933212 PMC8844144

[B14] Chung Y. H. Cai H. Steinmetz N. F. (2020). Viral Nanoparticles for Drug Delivery, Imaging, Immunotherapy, and Theranostic Applications. Adv. Drug Deliv. Rev. 156, 214–235. 10.1016/j.addr.2020.06.024 32603813 PMC7320870

[B15] Chung Y. H. Church D. Koellhoffer E. C. Osota E. Shukla S. Rybicki E. P. (2021). Integrating Plant Molecular Farming and Materials Research for Next-Generation Vaccines. Nat. Rev. Mater. 10.1038/s41578-021-00399-5 PMC8647509 34900343

[B16] Cordero T. Cerdán L. Carbonell A. Katsarou K. Kalantidis K. Daròs J.-A. (2017). Dicer-like 4 Is Involved in Restricting the Systemic Movement of Zucchini Yellow Mosaic Virus in Nicotiana Benthamiana. Mpmi 30, 63–71. 10.1094/MPMI-11-16-0239-R 27958768

[B17] Cordero T. Rosado A. Majer E. Jaramillo A. Rodrigo G. Daròs J.-A. (2018). Boolean Computation in Plants Using Post-translational Genetic Control and a Visual Output Signal. ACS Synth. Biol. 7, 2322–2330. 10.1021/acssynbio.8b00214 30212620

[B18] Cruz S. S. Chapman S. Roberts A. G. Roberts I. M. Prior D. A. Oparka K. J. (1996). Assembly and Movement of a Plant Virus Carrying a green Fluorescent Protein Overcoat. Proc. Natl. Acad. Sci. U.S.A. 93, 6286–6290. 10.1073/pnas.93.13.6286 8692807 PMC39014

[B19] De Coninck B. Verheesen P. Vos C. M. Van Daele I. De Bolle M. F. Vieira J. V. (2017). Fungal Glucosylceramide-specific Camelid Single Domain Antibodies Are Characterized by Broad Spectrum Antifungal Activity. Front. Microbiol. 8, 1–10. 10.3389/fmicb.2017.01059 28659884 PMC5469901

[B20] De Meyer T. Muyldermans S. Depicker A. (2014). Nanobody-based Products as Research and Diagnostic Tools. Trends Biotechnol. 32, 263–270. 10.1016/j.tibtech.2014.03.001 24698358

[B21] Dickmeis C. Honickel M. M. A. Fischer R. Commandeur U. (2015). Production of Hybrid Chimeric PVX Particles Using a Combination of TMV and PVX-Based Expression Vectors. Front. Bioeng. Biotechnol. 3, 1–12. 10.3389/fbioe.2015.00189 26636076 PMC4653303

[B22] Donini M. Marusic C. (2019). Current State-Of-The-Art in Plant-Based Antibody Production Systems. Biotechnol. Lett. 41, 335–346. 10.1007/s10529-019-02651-z 30684155

[B23] Edgue G. Twyman R. M. Beiss V. Fischer R. Sack M. (2017). Antibodies from Plants for Bionanomaterials. WIREs Nanomed Nanobiotechnol 9, e1462. 10.1002/wnan.1462 28345261

[B24] Fernández-Fernández M. R. Martínez-Torrecuadrada J. L. Roncal F. Domínguez E. García J. A. (2002). Identification of Immunogenic Hot Spots within Plum Pox Potyvirus Capsid Protein for Efficient Antigen Presentation. J. Virol. 76, 12646–12653. 10.1128/jvi.76.24.12646-12653.2002 12438590 PMC136723

[B25] Frías-Sánchez A. I. Quevedo-Moreno D. A. Samandari M. Tavares-Negrete J. A. Sánchez-Rodríguez V. H. González-Gamboa I. (2021). Biofabrication of Muscle Fibers Enhanced with Plant Viral Nanoparticles Using Surface Chaotic Flows. Biofabrication 13, 035015. 10.1088/1758-5090/abd9d7 33418551

[B26] Gal-On A. (2007). Zucchini Yellow Mosaic Virus: Insect Transmission and Pathogenicity - the Tails of Two Proteins. Mol. Plant Pathol. 8, 139–150. 10.1111/j.1364-3703.2007.00381.x 20507486

[B27] Ghannam A. Kumari S. Muyldermans S. Abbady A. Q. (2015). Camelid Nanobodies with High Affinity for Broad Bean Mottle Virus: a Possible Promising Tool to Immunomodulate Plant Resistance against Viruses. Plant Mol. Biol. 87, 355–369. 10.1007/s11103-015-0282-5 25648551

[B28] Gibson D. G. Young L. Chuang R.-Y. Venter J. C. Hutchison C. A. III Smith H. O. (2009). Enzymatic Assembly of DNA Molecules up to Several Hundred Kilobases. Nat. Methods 6, 343–345. 10.1038/nmeth.1318 19363495

[B29] Giritch A. Marillonnet S. Engler C. Van Eldik G. Botterman J. Klimyuk V. (2006). Rapid High-Yield Expression of Full-Size IgG Antibodies in Plants Coinfected with Noncompeting Viral Vectors. Proc. Natl. Acad. Sci. U.S.A. 103, 14701–14706. 10.1073/pnas.0606631103 16973752 PMC1566189

[B30] González-Gamboa I. Manrique P. Sánchez F. Ponz F. (2017). Plant-made Potyvirus-like Particles Used for Log-Increasing Antibody Sensing Capacity. J. Biotechnol. 254, 17–24. 10.1016/j.jbiotec.2017.06.014 28625680

[B31] Goulet M.-C. Gaudreau L. Gagné M. Maltais A.-M. Laliberté A.-C. Éthier G. (2019). Production of Biopharmaceuticals in Nicotiana Benthamiana-Axillary Stem Growth as a Key Determinant of Total Protein Yield. Front. Plant Sci. 10, 735. 10.3389/fpls.2019.00735 31244869 PMC6579815

[B32] Haldeman-Cahill R. Daròs J.-A. Carrington J. C. (1998). Secondary Structures in the Capsid Protein Coding Sequence and 3′ Nontranslated Region Involved in Amplification of the Tobacco Etch Virus Genome. J. Virol. 72, 4072–4079. 10.1128/jvi.72.5.4072-4079.1998 9557696 PMC109636

[B33] Hemmer C. Djennane S. Ackerer L. Hleibieh K. Marmonier A. Gersch S. (2018). Nanobody-mediated Resistance to Grapevine Fanleaf Virus in Plants. Plant Biotechnol. J. 16, 660–671. 10.1111/pbi.12819 28796912 PMC5787842

[B34] Houhou F. Martí M. Cordero T. Aragonés V. Sáez C. Cebolla‐Cornejo J. (2022). Carotenoid Fortification of Zucchini Fruits Using a Viral RNA Vector. Biotechnol. J., e2100328. 10.1002/biot.202100328 35157358

[B35] Juarez P. Virdi V. Depicker A. Orzaez D. (2016). Biomanufacturing of Protective Antibodies and Other Therapeutics in Edible Plant Tissues for Oral Applications. Plant Biotechnol. J. 14, 1791–1799. 10.1111/pbi.12541 26873071 PMC5067594

[B36] Julve Parreño J. M. Huet E. Fernández-del-Carmen A. Segura A. Venturi M. Gandía A. (2018). A Synthetic Biology Approach for Consistent Production of Plant-Made Recombinant Polyclonal Antibodies against Snake Venom Toxins. Plant Biotechnol. J. 16, 727–736. 10.1111/pbi.12823 28850773 PMC5814581

[B37] Kelloniemi J. Mäkinen K. Valkonen J. P. T. (2008). Three Heterologous Proteins Simultaneously Expressed from a Chimeric Potyvirus: Infectivity, Stability and the Correlation of Genome and Virion Lengths. Virus. Res. 135, 282–291. 10.1016/j.virusres.2008.04.006 18511144

[B38] Kim J. H. Lee S.-R. Li L.-H. Park H.-J. Park J.-H. Lee K. Y. (2011). High Cleavage Efficiency of a 2A Peptide Derived from Porcine Teschovirus-1 in Human Cell Lines, Zebrafish and Mice. PLoS One 6, e18556. 10.1371/journal.pone.0018556 21602908 PMC3084703

[B39] Le D. H. T. Commandeur U. Steinmetz N. F. (2019). Presentation and Delivery of Tumor Necrosis Factor-Related Apoptosis-Inducing Ligand via Elongated Plant Viral Nanoparticle Enhances Antitumor Efficacy. ACS Nano 13, 2501–2510. 10.1021/acsnano.8b09462 30668110

[B40] Lico C. Benvenuto E. Baschieri S. (2015). The Two-Faced Potato Virus X: From Plant Pathogen to Smart Nanoparticle. Front. Plant Sci. 6, 1–8. 10.3389/fpls.2015.01009 26635836 PMC4646960

[B41] Lomonossoff G. P. D’Aoust M.-A. (2016). Plant-produced Biopharmaceuticals: A Case of Technical Developments Driving Clinical Deployment. Science 353, 1237–1240. 10.1126/science.aaf6638 27634524

[B42] Lomonossoff G. P. Wege C. (2018). TMV Particles: The Journey from Fundamental Studies to Bionanotechnology Applications. Adv. Virus. Res. 102, 149–176. 10.1016/bs.aivir.2018.06.003 30266172 PMC7112118

[B43] Majer E. Llorente B. Rodríguez-Concepción M. Daròs J.-A. (2017). Rewiring Carotenoid Biosynthesis in Plants Using a Viral Vector. Sci. Rep. 7, 1–10. 10.1038/srep41645 28139696 PMC5282570

[B44] Majer E. Navarro J. A. Daròs J. A. (2015). A Potyvirus Vector Efficiently Targets Recombinant Proteins to Chloroplasts, Mitochondria and Nuclei in Plant Cells when Expressed at the Amino Terminus of the Polyprotein. Biotechnol. J. 10, 1792–1802. 10.1002/biot.201500042 26147811

[B45] Malaquias A. D. M. Marques L. E. C. Pereira S. S. de Freitas Fernandes C. Maranhão A. Q. Stabeli R. G. (2021). A Review of Plant-Based Expression Systems as a Platform for Single-Domain Recombinant Antibody Production. Int. J. Biol. Macromolecules 193, 1130–1137. 10.1016/j.ijbiomac.2021.10.126 34699899

[B46] Manuel-Cabrera C. A. Vallejo-Cardona A. A. Padilla-Camberos E. Hernández-Gutiérrez R. Herrera-Rodríguez S. E. Gutiérrez-Ortega A. (2016). Self-assembly of Hexahistidine-Tagged Tobacco Etch Virus Capsid Protein into Microfilaments that Induce IgG2-specific Response against a Soluble Porcine Reproductive and Respiratory Syndrome Virus Chimeric Protein. Virol. J. 13, 1–6. 10.1186/s12985-016-0651-y 27894314 PMC5126848

[B47] Marsian J. Lomonossoff G. P. (2016). Molecular Pharming - VLPs Made in Plants. Curr. Opin. Biotechnol. 37, 201–206. 10.1016/j.copbio.2015.12.007 26773389

[B48] Martí M. Diretto G. Aragonés V. Frusciante S. Ahrazem O. Gómez-Gómez L. (2020). Efficient Production of Saffron Crocins and Picrocrocin in Nicotiana Benthamiana Using a Virus-Driven System. Metab. Eng. 61, 238–250. 10.1016/j.ymben.2020.06.009 32629020

[B49] Mitchell L. S. Colwell L. J. (2018). Comparative Analysis of Nanobody Sequence and Structure Data. Proteins 86, 697–706. 10.1002/prot.25497 29569425 PMC6033041

[B50] Muyldermans S. (2013). Nanobodies: Natural Single-Domain Antibodies. Annu. Rev. Biochem. 82, 775–797. 10.1146/annurev-biochem-063011-092449 23495938

[B51] Oliveira S. Heukers R. Sornkom J. Kok R. J. Van Bergen En Henegouwen P. M. P. (2013). Targeting Tumors with Nanobodies for Cancer Imaging and Therapy. J. Controlled Release 172, 607–617. 10.1016/j.jconrel.2013.08.298 24035975

[B52] Ortega-Rivera O. A. Shukla S. Shin M. D. Chen A. Beiss V. Moreno-Gonzalez M. A. (2021). Cowpea Mosaic Virus Nanoparticle Vaccine Candidates Displaying Peptide Epitopes Can Neutralize the Severe Acute Respiratory Syndrome Coronavirus. ACS Infect. Dis. 7, 3096–3110. 10.1021/acsinfecdis.1c00410 34672530

[B53] Peyret H. Gehin A. Thuenemann E. C. Blond D. El Turabi A. Beales L. (2015). Tandem Fusion of Hepatitis B Core Antigen Allows Assembly of Virus-like Particles in Bacteria and Plants with Enhanced Capacity to Accommodate Foreign Proteins. PLoS One 10, e0120751. 10.1371/journal.pone.0120751 25830365 PMC4382129

[B54] Peyret H. Lomonossoff G. P. (2015). When Plant Virology metAgrobacterium: the Rise of the Deconstructed Clones. Plant Biotechnol. J. 13, 1121–1135. 10.1111/pbi.12412 26073158 PMC4744784

[B55] Peyret H. Steele J. F. C. Jung J.-W. Thuenemann E. C. Meshcheriakova Y. Lomonossoff G. P. (2021). Producing Vaccines against Enveloped Viruses in Plants: Making the Impossible, Difficult. Vaccines 9, 780. 10.3390/vaccines9070780 34358196 PMC8310165

[B56] Revers F. García J. A. (2015). Molecular Biology of Potyviruses. Adv. Virus. Res. 92, 101–199. 10.1016/bs.aivir.2014.11.006 25701887

[B57] Röder J. Dickmeis C. Commandeur U. (2019). Small, Smaller, Nano: New Applications for Potato Virus X in Nanotechnology. Front. Plant Sci. 10, 158. 10.3389/fpls.2019.00158 30838013 PMC6390637

[B58] Röder J. Dickmeis C. Fischer R. Commandeur U. (2018). Systemic Infection of Nicotiana Benthamiana with Potato Virus X Nanoparticles Presenting a Fluorescent iLOV Polypeptide Fused Directly to the Coat Protein. Biomed. Res. Int. 2018, 1–12. 10.1155/2018/9328671 PMC5831704 29662905

[B59] Röder J. Fischer R. Commandeur U. (2017). Adoption of the 2A Ribosomal Skip Principle to Tobacco Mosaic Virus for Peptide Display. Front. Plant Sci. 8, 1–11. 10.3389/fpls.2017.01125 28702043 PMC5487473

[B60] Rybicki E. P. (2020). Plant Molecular Farming of Virus‐like Nanoparticles as Vaccines and Reagents. WIREs Nanomed Nanobiotechnol 12, 1–22. 10.1002/wnan.1587 31486296

[B61] Sainsbury F. Cañizares M. C. Lomonossoff G. P. (2010). Cowpea Mosaic Virus: The Plant Virus-Based Biotechnology Workhorse. Annu. Rev. Phytopathol. 48, 437–455. 10.1146/annurev-phyto-073009-114242 20455698

[B62] Salema V. Marín E. Martínez-Arteaga R. Ruano-Gallego D. Fraile S. Margolles Y. (2013). Selection of Single Domain Antibodies from Immune Libraries Displayed on the Surface of *E. coli* Cells with Two β-Domains of Opposite Topologies. PLoS One 8, e75126. 10.1371/journal.pone.0075126 24086454 PMC3781032

[B63] Salvador J.-P. Vilaplana L. Marco M.-P. (2019). Nanobody: Outstanding Features for Diagnostic and Therapeutic Applications. Anal. Bioanal. Chem. 411, 1703–1713. 10.1007/s00216-019-01633-4 30734854

[B64] Sánchez F. Sáez M. Lunello P. Ponz F. (2013). Plant Viral Elongated Nanoparticles Modified for Log-Increases of Foreign Peptide Immunogenicity and Specific Antibody Detection. J. Biotechnol. 168, 409–415. 10.1016/j.jbiotec.2013.09.002 24055625

[B65] Satheeshkumar P. K. (2020). Expression of Single Chain Variable Fragment (scFv) Molecules in Plants: A Comprehensive Update. Mol. Biotechnol. 62, 151–167. 10.1007/s12033-020-00241-3 32036549 PMC7091320

[B66] Schmidt T. G. M. Batz L. Bonet L. Carl U. Holzapfel G. Kiem K. (2013). Development of the Twin-Strep-Tag and its Application for Purification of Recombinant Proteins from Cell Culture Supernatants. Protein Expr. Purif. 92, 54–61. 10.1016/j.pep.2013.08.021 24012791

[B67] Shukla S. DiFranco N. A. Wen A. M. Commandeur U. Steinmetz N. F. (2015). To Target or Not to Target: Active vs. Passive Tumor Homing of Filamentous Nanoparticles Based on Potato Virus X. Cel. Mol. Bioeng. 8, 433–444. 10.1007/s12195-015-0388-5 PMC4540758 26316894

[B68] Shukla S. Hu H. Cai H. Chan S.-K. Boone C. E. Beiss V. (2020a). Plant Viruses and Bacteriophage-Based Reagents for Diagnosis and Therapy. Annu. Rev. Virol. 7, 559–587. 10.1146/annurev-virology-010720-052252 32991265 PMC8018517

[B69] Shukla S. Roe A. J. Liu R. Veliz F. A. Commandeur U. Wald D. N. (2020b). Affinity of Plant Viral Nanoparticle Potato Virus X (PVX) towards Malignant B Cells Enables Cancer Drug Delivery. Biomater. Sci. 8, 3935–3943. 10.1039/d0bm00683a 32662788 PMC7810362

[B70] Shukla S. Steinmetz N. F. (2016). Emerging Nanotechnologies for Cancer Immunotherapy. Exp. Biol. Med. (Maywood) 241, 1116–1126. 10.1177/1535370216647123 27190253 PMC4950359

[B71] Simmons H. E. Dunham J. P. Zinn K. E. Munkvold G. P. Holmes E. C. Stephenson A. G. (2013). Zucchini Yellow Mosaic Virus (ZYMV, Potyvirus): Vertical Transmission, Seed Infection and Cryptic Infections. Virus. Res. 176, 259–264. 10.1016/j.virusres.2013.06.016 23845301 PMC3774540

[B72] Smolenska L. Roberts I. M. Learmonth D. Porter A. J. Harris W. J. Wilson T. M. A. (1998). Production of a Functional Single Chain Antibody Attached to the Surface of a Plant Virus. FEBS Lett. 441, 379–382. 10.1016/S0014-5793(98)01586-5 9891975

[B73] Stander J. Chabeda A. Rybicki E. P. Meyers A. E. (2021). A Plant-Produced Virus-like Particle Displaying Envelope Protein Domain III Elicits an Immune Response against West Nile Virus in Mice. Front. Plant Sci. 12, 1–12. 10.3389/fpls.2021.738619 PMC8475786 34589108

[B74] Steele J. F. C. Peyret H. Saunders K. Castells‐Graells R. Marsian J. Meshcheriakova Y. (2017). Synthetic Plant Virology for Nanobiotechnology and Nanomedicine. WIREs Nanomed Nanobiotechnol 9, 1–18. 10.1002/wnan.1447 PMC5484280 28078770

[B75] Steinmetz N. F. Manchester M. (2016). “Viral Nanoparticles: Tools for Materials Science and Biomedicine,” in Handbook Of Clinical Nanomedicine: Nanoparticles, Imaging, Therapy And Clinical Applications (Singapore: Pan Stanford Publishing Pte. Ltd.), 641–656. 10.4032/9789814669214

[B76] Thole V. Worland B. Snape J. W. Vain P. (2007). The pCLEAN Dual Binary Vector System for Agrobacterium-Mediated Plant Transformation. Plant Physiol. 145, 1211–1219. 10.1104/pp.107.108563 17932303 PMC2151721

[B77] Thuenemann E. C. Le D. H. T. Lomonossoff G. P. Steinmetz N. F. (2021). Bluetongue Virus Particles as Nanoreactors for Enzyme Delivery and Cancer Therapy. Mol. Pharmaceutics 18, 1150–1156. 10.1021/acs.molpharmaceut.0c01053 PMC8086382 33566625

[B78] Tusé D. Nandi S. McDonald K. A. Buyel J. F. (2020). The Emergency Response Capacity of Plant-Based Biopharmaceutical Manufacturing-What it Is and what it Could Be. Front. Plant Sci. 11, 594019. 10.3389/fpls.2020.594019 33193552 PMC7606873

[B79] Uhde‐Holzem K. McBurney M. Tiu B. D. B. Advincula R. C. Fischer R. Commandeur U. (2016). Production of Immunoabsorbent Nanoparticles by Displaying Single‐Domain Protein A on Potato Virus X. Macromol. Biosci. 16, 231–241. 10.1002/mabi.201500280 26440117

[B80] Uhde-Holzem K. Schlösser V. Viazov S. Fischer R. Commandeur U. (2010). Immunogenic Properties of Chimeric Potato Virus X Particles Displaying the Hepatitis C Virus Hypervariable Region I Peptide R9. J. Virol. Methods 166, 12–20. 10.1016/j.jviromet.2010.01.017 20138085

[B81] Uranga M. Aragonés V. Selma S. Vázquez‐Vilar M. Orzáez D. Daròs J. A. (2021a). Efficient Cas9 Multiplex Editing Using Unspaced sgRNA Arrays Engineering in a Potato Virus X Vector. Plant J. 106, 555–565. 10.1111/tpj.15164 33484202 PMC8251967

[B82] Uranga M. Vazquez-Vilar M. Orzáez D. Daròs J.-A. (2021b). CRISPR-Cas12a Genome Editing at the Whole-Plant Level Using Two Compatible RNA Virus Vectors. CRISPR J. 4, 761–769. 10.1089/crispr.2021.0049 34558964

[B83] Wang M. Gao S. Zeng W. Yang Y. Ma J. Wang Y. (2020). Plant Virology Delivers Diverse Toolsets for Biotechnology. Viruses 12, 1338. 10.3390/v12111338 PMC7700544 33238421

[B84] Wang W. Yuan J. Jiang C. (2021). Applications of Nanobodies in Plant Science and Biotechnology. Plant Mol. Biol. 105, 43–53. 10.1007/s11103-020-01082-z 33037986 PMC7547553

[B85] Wang Y. Fan Z. Shao L. Kong X. Hou X. Tian D. (2016). Nanobody-derived Nanobiotechnology Tool Kits for Diverse Biomedical and Biotechnology Applications. Ijn 11, 3287–3303. 10.2147/IJN.S107194 27499623 PMC4959585

[B86] Yusibov V. Kushnir N. Streatfield S. J. (2016). Antibody Production in Plants and Green Algae. Annu. Rev. Plant Biol. 67, 669–701. 10.1146/annurev-arplant-043015-111812 26905655

[B87] Yuste-Calvo C. López-Santalla M. Zurita L. Cruz-Fernández C. F. Sánchez F. Garín M. I. (2019). Elongated Flexuous Plant Virus-Derived Nanoparticles Functionalized for Autoantibody Detection. Nanomaterials 9, 1438. 10.3390/nano9101438 PMC6835482 31658770

