# Supplementary material for: Production of Potyvirus-Derived Nanoparticles Decorated with a Nanobody in Biofactory Plants
Source: Front Bioeng Biotechnol. 2022 Mar 31;10:877363. doi: 10.3389/fbioe.2022.877363 (PMC9008781; doi:10.3389/fbioe.2022.877363)
Supplement: Supplementary file 1 [file DataSheet1.PDF]

SUPPLEMENTARY MATERIAL

Production of Potyvirus-Derived Nanoparticles Decorated with a Nanobody in Biofactory Plants

Maricarmen Martí, Fernando Merwaiss, Anamarija Butković, and José-Antonio Daròs*

Instituto de Biología Molecular y Celular de Plantas (Consejo Superior de Investigaciones Científicas – Universitat Politècnica de València), 46022 Valencia, Spain

***Correspondence**:

José-Antonio Daròs

jadaros@ibmcp.upv.es

Figure S1. Nucleotide sequences of ZYMV-wt (GenBank accession number KX499498) and the derived recombinant viruses ZYMVΔ, -αGFP, and αGFP-F2A. The boundaries of viral cistrons are indicated on blue background. **Start** and **stop** codons are underlined. Heterologous sequences were inserted between nucleotide positions **8550** and **8642** (underlined). cDNAs corresponding to the anti-green fluorescent protein (**αGFP**) nanobody, and pircornavirus **F2A** peptides are in green and blue, respectively.

>ZYMV-wt
AAATTAAAACAAATCACAAAGACTACAGAAATCAACGAACAAACAAACGAATTTTAAACCGTGTTAACAAACAAG
CAATTTATAATTCGCACAGCATCAAGAATTTCTGCAATCATTTTGTTTATTTTAGACACAACAATGGCCTCAGTT
ATGATTGGTTCAATCTCCGTACCCATCGCACAGCCTGCGCAGTGTGCAAACACCCAAGCGAGCAACCGGGTTAAC
GTAGTGGCACCTGGCCACATGGCAACATGCCCACCATCATTGAAAACGCACACATACTACATGCATGAGTCTAAG
AAGTTGATAAATTCAGATAAAAGCAATGAAATTTTGAACAATTTCTTTAACACTGATGAGATGAAATTCGCGCTC
ACTAGGAATGAAATGAGTAAGGTGAAAAAGGGTCCGAATGGGAGGGTAGTTCTTCGCAAACCGAGCAAGCAGCGG
GTTTTCGCTCGGATCGAGCAGGATGAGGCAGCACGCAAGGAAGAAGCTGTTTTCCTTGCAGGAAATTATGATGGT
TCGATCACAAATCTAGTGAGTGTTCTTCCATCTGAAATGACTCGCGATGTTGATGCGAGTTTGCGATCACCATTT
TACAAGCGCACATATAAGAAGGACAGAAAGAAAGTGGCGCAAAAGCAAATCGCGCAGGCACCACTTAACAGCTTG
TGCACACGTGTTCTTAAAATTGCGCGCAATAGAAACATCCCTGTTGAGATAATTGGCAACAAGAAAGCAAGACAT
ACACTTACCTTCAAGAGGTTTAGGGGATGTTTTGTTGGAAAGGTGTCAGTTGCACATGAGGAAGGACGAATGCAA
CACACTGAGATATCATACGAGCAGTTTGAATGGATTCTACAAGCTATTTGTCGGGTTACTTATACAGAGCGAATT
CGTGAGGAAGACATTAAACCAGGTTGTAGTGGGTGGGTGTTAGGCACTGATCATACATTGACCAAGAGATATTCA
AGATTGCCACATCTGGTAATTCGAGGTAGAGATGATGACGGGATTGTGAACGCGCTGGAACCGGTGTTATTTTAC
AGCGAAGTTGACCACTATTCGTCGCAACCGGAAGTTCAGTTCTTCCAAGGATGGCGACGAATGTTCGACAAGTTT
AGACCCAGTCCAGATCATGTGTGCAAAGTTGATCACAACAATGAGGAGTGTGGTGAGTTAGCAGCAATCTTTTGT
CAGGCTTTGTTCCCAGTAGTGAAATTATCGTGCCAAACATGCAGAGAAAAGCTTAGTAGAGTTAGCTTCGAGGAA
TTCAAAGATTCTTTGAATACAAACTTTATTATCCATAAGGACGAATGGGATAGTTTCAAAGAGGGCTCTCATTAC
GATAATATTTTCAAATTAATTAAAGTAGCAACACAGGTAACTCAGAATCTCAAGCTCTCATCTGAAGTAATGAAG
TTAGTTCAGAACCACACAAGCACTCACATGAAGCAAATACAAGATATCAACAAGGCGCTCATGAAAGGTTCATTG
GTTACGCAAGACGAATTGGACTTGGCTTTGAAACAGCTTCTAGAAATGACTCAGTGGTTTAAGAACCACATGCAC
CTGACTGGTGAAGAAGCATTGAAGATGTTCAGAAACAAGCGCTCTAGCAAGGCTATGATAAATCCTAACCTTTTA

TGTGATAACCAGTTGGACAAAAATGGAAACTTTGTCTGGGGAGAAAGAGGATATCATTCCAAGCGATTATTCAAG
AACTTCTTTGAGGAAGTTATACCAAGTGAAGGATATACGAAATACGTAGTGCGAAACTTCCCAAATGGTACTCGT
AAGTTGGCCATAGGCTCATTGATCGTACCACTCAACTTGGATAGGGCACGCACTGCACTCCTTGGAGAGAGTATT
GAGAAGAAGCCACTCACATCAGCATGTGTCTCCCAACAGAATGGAAATTATATACACTCATGCTGCTGTGTGACG
ATGGATGATGGAACTCCGATGTACTCAGAGCTTAAGAGCCCGACGAAGAGACATCTAGTTATAGGAGCTTCTGGT
GATCCAAAGTACATTGATTTACCAGCATCTGAGGCAGAACGCATGTATATAGCAAAGGAAGGTTATTGCTATCTT
AACATTTTTCTCGCAATGCTTGTGAATGTTAATGAGAACGAGGCAAAGGATTTCACCAAAATGATTCGTGATGTT
TTGATCCCTATGCTTGGGCAATGGCCTTCGTTGATGGATGTCGCGACTGCAGCATACATTTTAGGTGTATTCCAT
CCTGAAACGCGATGCGCTGAATTACCTAGGATTCTTGTTGACCATGCTACGCAAACCATGCATGTCATTGATTCA
TATGGATCATTAACTGTTGGTTATCACGTGCTCAAGGCCGGAACTGTCAATCATTTAATTCAATTTGCCTCAAAT
GATCTGCAGAGCGAGATGAAGCATTACAGAGTCGGCGGAACGCCAACACAGCGCATAAAACTTGAGGAGCAATTG
ATTAAAGGAATCTTCAAACCAAAACTTATGATGCAGCTCTTGCACGATGACCCATACATATTATTGCTTGGCATG
ATCTCACCCACCATTCTTGTGCACATGTATAGGATGCGTCATTTTGAGCGAGGTATTGAAATATGGATTAAGAGA
GATCATGAAATTGGAAAGATTTTCGTCATATTGGAACAGCTCACACGGAAGGTTGCTCTGGCTGAAGTTCTTGTT
GATCAGCTCGATTTGATAAGTGAAGCTTCACCACATTTACTTGAAATCATGAAGGGTTGTCAAGATAATCAAAGG
GCGTATGTACCTGCGCTGGATCTGTTAACGATACAAGTAGAGCGTGAGTTTTCAAATAAAGAACTTAAAACCAAT
GGCTATCCAGATTTGCAGCAAACGTTGTTTGATATGAGAGAAAAAATGTATGCAAAGCAGTTGCACAGTTCATGG
CAAGAGCTAAGCTTGCTGGAAAAATCTTGTGTAACCGTGCGATTGAAGCAATTCTCGATTTTTACGGAAAGAAAT
TTAATCCAGCGAGCAAAAGAAGGAAAGCGCACATCTTCGCTACAATTTGTTCACGAGTGCTTTATCACGACCCGA
GTACATGCGAAGAGCATTCGCGATGCAGGCGTGCGCAAGCTAAATGAGGCTCTCGTTGGAACTTGTAAGTTCTTT
TTCTCTTGTGGTTTCAAGATTTTTGCGCGGTGTTACAGCGACATTATATACCTTGTGAACGTGTGTTTGGTATTC
TCTTTGGTGTTACAAATGTCTAATACTGTGCGCAACATGATAGCGGCGACAAGGGAAGAAAAGGAGAGAGCGATG
GCAAATAAAGCTGATGAAAATGAAAGGACGTTAATGCACATGTACCACATTTTCAGTAAGAAGCAGGATGAAGCG
CCCATATATAACGACTTTCTTGAACATGTTCGCAATGTAAGACCAGATCTTGAGGAAACCCTCTTGTACATGGCT
GGTGCAGAGGTTGTTGCAACACAGGCCAAGTCAGCGGTTCAGATTCAGTTCGAGAAGATTATAGCTGTATTGGCG
CTGCTCACTATGTGCTTTGACGCCGAAAGAAGTGATGCCATTTTCAAGATTTTGACAAAGCTCAAAACAGTTTTT
GGCACGGTTGGAGAAACGGTCCGACTTCAAGGACTTGAAGATATTGAGAGCTTGGAAGACGATAAGAGACTCACA
ATTGACTTTGATATTAACACGAATGAGGCTCAATCGTCGACAACGTTTGATGTTCATTTTGATGATTGGTGGAAT
CGGCAGCTACAGCAAAATCGCACAGTTCCACATTATAGGACCACAGGTAAATTCCTCGAATTCACCAGAAGCACT
GCAGCTTTTGTGGCTAATGAAATAGCATCATCAAGTGAAGGAGAATTTTTAGTTAGAGGAGCAGTGGGTTCTGGA
AAATCAACGAGCTTGCCTGCGCATCTTTCCAAGAAGGGTAAAGTATTACTACTCGAACCCACACGCCCTTTGGCG
GAGAATGTCAGTAGACAGTTGGCAGGCGATCCTTTTTTCCAAAACGTCACACTCAGAATGAGAGGGCTAAATTGC
TTTGGTTCAAGTAACATTACAGTGATGACGAGTGGATTTGCTTTTCACTACTATGTTAATAATCCACACCAATTA
ATGGAATTTGACTTTATTATCATAGACGAATGCCATGTTACGGACAGTGCGACTATAGCTTTCAATTGCGCACTT
AAGGAGTATAATTTTGCTGGCAAATTGATTAAAGTATCTGCAACGCCGCCAGGGAGGGAGTGTGATTTCGATACG
CAATTCGCGGTGAAAGTCAAAACGGAGGACCACCTTTCATTCCATGCATTCGTTGGCGCACAGAAGACTGGTTCA
AACGCTGATATGGTTCAGCATGGCAATAACATACTTGTGTATGTTGCAAGTTATAACGAAGTAGACATGCTTTCC
AAATTACTCACTGAGCGACAATTTTCAGTGACGAAGGTAGATGGGCGAACAATGCAACTTGGGAAAACTACCATT
GAAACGCATGGCACTAGTCAAAAGCCTCATTTCATAGTAGCCACAAACATCATTGAGAATGGAGTGACGTTGGAT
GTCGAATGTGTTGTTGATTTTGGGCTAAAAGTGGTCGCAGAATTAGACAGCGAAAATCGGTGTGTGCGCTACAAC
AAGAAATCAGTTAGTTATGGAGAAAGGATTCAGCGGCTAGGGAGAGTGGGGAGATCTAAGCCTGGAACTGCATTG
CGTATAGGGCACACAGAAAAAGGCATCGAGAACATTCCCGAATTCATTGCCACAGAAGCAGCAGCCTTATCATTT
GCATATGGGCTTCCAGTCACCACGCATGGGGTTTCCACAAATATACTCGGAAAGTGCACAGTTAAACAGATGAGA
TGTGCTTTGAATTTTGAGTTAACTCCTTTCTTTACCACTCATTTAATTCGCCATGATGGTAGCATGCACCCACTG
ATACACGAAGAACTGAAACAATTTAAACTCAGGGACTCAGAAATGGTGCTCAACAAGGTTGCATTACCTCACCAA
TTTGTGAGTCAATGGTTGGATCAAAGTGAGTATGAACGCATTGGAGTGCACGTTCAATGCCATGAGAGCACACGC
ATACCTTTTTACACAAATGGAGTGCCTGATAAAGTCTATGAGAAAATTTGGAAGTGCATACAAGAAAACAAGAAT
GATGCGGTTTTTGGTAAGCTTTCAAGTGCTTGTTCGACTAAGGTTAGTTATACACTCAGCACTGATCCAGCAGCA
TTACCCAGAACTATTGCAATCATCGACCACCTGCTTGCCGAGGAAATGATGAAGCGGAATCACTTCGACACGATC
AGCTCAGCTGTAACGGGCTATTCATTTTCCCTCGCTGGAATTGCTGATTCTTTTAGGAAAAGGTATATGCGCGAT
TACACAGCGCATAACATTGCAATTCTCCAACAAGCACGTGCCCAGCTGCTCGAATTCAATAGTAAAAATGTGAAC
ATCAACAACCTGTCCGATCTGGAAGGAATTGGAGTTATTAAGTCGGTGGTGTTGCAAAGTAAGCAAGAGGTCAGC
AACTTCCTAGGACTTCGCGGTAAATGGGATGGGCGGAAATTTGCGAATGATGTGATATTGGCGATCATGACACTC
TTAGGAGGTGGATGGTTCATGTGGGAATACTTCACGAAAAAGATCAATGAACCCGTGCGCGTTGAAAGCAAGAAA
CGGCGATCTCAAAAGTTGAAATTCAGGGATGCGTACGATAGGAAAGTCGGACGTGAGATTTTTGGCGATGATGAC
ACAATTGGGCGCACTTTTGGCGAAGCTTACACGAAGAGAGGAAAGGTCAAAGGAAACAACAGCACAAAAGGAATG
GGACGGAAAACTCGCAATTTTGTGCATTTATATGGTGTGGAGCCTGAGAATTACAGCTTTATTAGATTTGTGGAC

CCTCTCACTGGCCATACATTGGACGAAAGCACCCATACAGACATTTCGTTAGTGCAGGAGGAGTTTGGAAGTATT
AGAGAGAAATTTCTGGAGAATGATTTAATCTCGAGGCAGTCTATTATTAACAAACCCGGTATTCAAGCATATTTT
ATGGGCAAGGGCACCGAAGAAGCACTCAAAGTTGATTTGACTCCTCATGTACCATTGCTTCTGTGCAGAAACACC
AATGCCATTGCGGGGTATCCAGAGAGAGAAAATGAGTTGAGACAAACCGGCACACCAGTTAAGGTTTCTTTTAAA
GACGTGCCAGAGAAAAACGAACATGTCGAGTTGGAGAGCAAATCCATCTACAAAGGAGTGCGCGATTACAATGGC
ATTTCAACAATCGTCTGTCAATTAACGAACGATTCTGATGGCCTCAAGGAGACTATGTATGGTATTGGCTACGGG
CCGATAATCATTACTAATGGGCACCTCTTCAGGAAAAACAATGGTACACTTCTAGTCAGGTCTTGGCATGGTGAA
TTCACTGTTAAAAATACCACAACGCTCAAAGTGCATTTCATAGAAGGGAAGGATGTTGTTTTAGTGCGTATGCCA
AAGGACTTTCCACCGTTCAAAAGCAACGCTTCTTTTAGAGCGCCAAAACGCGAGGAACGAGCATGCTTGGTTGGA
ACAAATTTTCAAGAGAAGAGTCTCCGCTCCACTGTTTCAGAATCTTCAATGACAATACCTGAAGGAACTGGCTCA
TATTGGATTCATTGGATTTCAACCAATGAAGGGGACTGCGGATTACCCATGGTTTCAACAACGGATGGTAAGATA
ATTGGAGTTCATGGTTTGGCTTCCACAGTCTCATCTAAGAATTATTTTGTCCCATTCACTGATGATTTTATAGCC
ACGCATTTGAGCAAGCTTGATGATCTCACATGGACTCAGCATTGGCTATGGCAACCTAGCAAAATCGCGTGGGGA
ACGCTCAACTTAGTTGATGAACAACCAGGGCCTGAATTTCGTATTTCAAATCTAGTCAAGGATTTGTTCACTTCT
GGTGTTGAAACACAGAGCAAGCGGGAAAGATGGGTCTACGAAAGCTGTGAAGGGAACCTTCGAGCTGTTGGAACT
GCGCAATCAGCGTTAGTCACCAAACATGTTGTCAAGGGCAAGTGTTCTTTCTTCGAAGAATATTTGCAAACACAC
GCAGAAGCGAGCGCCTATTTCAGACCCTTAATGGGAGAGTACCAGCCGAGCAAGTTGAACAAAGAGGCCTTCAAA
AAGGATTTCTTTAAATACAACAAACCCGTCACTGTTAATCAATTGGATCATGATAAATTTTTAGAAGCAGTGGAT
GGGGTTATACGTATGATGTGTGACTTTGAGTTCAATGAATGCCGATTCATTACAGATCCCGAGGAAATTTACAAC
TCTCTGAACATGAAAGCAGCAATTGGAGCCCAATATAGAGGAAAGAAGAAAGAATATTTTGAAGGGCTAGATGAT
TTTGATCGAGAGCGACTATTATTTCAAAGTTGTGAAAGGTTGTTTAATGGCTATAAAGGTTTGTGGAATGGATCT
TTAAAGGCTGAGCTCAGGCCGCTTGAGAAAGTCAGGGCTAACAAAACACGAACTTTTACAGCAGCGCCAATTGAT
ACATTGCTTGGAGCTAAAGTTTGCGTGGATGATTTCAATAATGAATTTTACAGCAAAAATCTCAAGTGTCCATGG
ACGGTTGGCATGACAAAATTTTATGGTGGTTGGGATAAATTGATGAGATCGTTACCTGATGGTTGGTTATACTGT
CATGCTGATGGATCACAGTTTGACAGTTCATTAACCCCAGCTCTATTGAATGCAGTTCTTATAATCAGGTCTTTT
TACATGGAAGATTGGTGGGTTGGCCAAGAGATGCTCGAAAATCTCTATGCTGAGATTGTGTACACTCCAATTCTT
GCTCCGGATGGAACAATTTTCAAGAAATTTAGAGGTAACAACAGTGGGCAACCCTCAACAGTGGTGGATAACACA
CTAATGGTTGTGATCTCTATTTACTATGCGTGCATGAAGTTTGGGTGGAATTGCGAGGAAATTGAGAATAAACTT
GTCTTCTTTGCAAATGGAGATGACCTGATACTTGCAGTCAGAGATGAAGACAGCGGCTTACTTGATAACATGTCA
TCCTCTTTTTCCGAACTTGGACTGAATTACGATTTTTCGGAACGCACGCACAAAAGAGAAGATCTTTGGTTCATG
TCCCACCAGGCAATGTTAATTGATGGAATGTACATCCCAAAACTTGAGAAAGAGAGAATTGTTTCAATTCTAGAA
TGGGATAGAAGTAAGGAAATAATGCACCGAACAGAGGCTATTTGCGCTGCAATGATTGAAGCATGGGGACACACC
GAGCTTTTACAAGAAATCAGAAAGTTTTACCTGTGGTTCGTTGAAAAGGAAGAAGTGCGAGAATTGGCTGCCCTC
GGAAAAGCTCCATACATAGCTGAGACAGCTCTTCGTAAGTTATACACTGACAAGGGAGCGGATACGAGTGAACTG
GCACGCTATCTACGAGCCCTCCATCAAGATATCTTCTTTGAACAAGGAGACACTGTAATGCTCCAATCAGGCACT
CAGCCAACTGTGGCAGACGCTGGGGCTACAAAGAAAGACAAAGAAGATGACAAAGGGAAAAACAAGGATGTTTCA
GGCTCCGGCTCAGGTGAGAAAACGATAGCAGCTGTCACAAAGGACAAGGATGTGAATGCTGGTTCTCATGGGAAG
ATCGTGCCGCGTCTTTCAAAGATCACAAAGAAAATGTCACTGCCACGCGTGAAAGGAAATGTGATACTCGATATC
GATCATTTGCTGGAATATAAACCGGATCAAATCGAGTTGTACAACACACGAGCGTCTCATCAGCAATTCGCTTCC
TGGTTTAACCAAGTTAAAACAGAATATGATCTGAATGAGCAACAGATGGGAGTTGTAATGAATGGTTTCATGGTT
TGGTGTATTGAAAATGGCACCTCACCCGACATTAATGGAGTGTGGGTTATGATGGACGGAAATGAGCAAGTTGAA
TATCCTTTGAAACCAATAGTTGAAAATGCAAAGCCAACGCTGCGGCAAATAATGCATCATTTTTCAGATGCAGCG
GAGGCATACATAGAGATGAGAAATGCAGAGGCACCATACATGCCGAGGTATGGTTTGCTTCGAAACCTACGGGAT
AGGAGTTTGGCACGATATGCTTTCGATTTCTATGAAGTCAATTCTAAAACTCCTGAAAGAGCCCGTGAAGCTGTT
GCGCAAATGAAAGCAGCAGCTCTTAGCAATGTTTCTTCAAGGTTGTTTGGCCTTGATGGAAATGTTGCCACTACT
AGCGAAGACACTGAACGGCACACTGCACGTGATGTTAATAGAAACATGCACACCTTACTAGGTGTGAATACAATG
CAGTAAAGGGTAGGTCGCCTACCTAGGTTATTGTTTCGCTGCCGACGTAATTCTAATATTTACCGCTTTATTTGA
TATCTTTAAATTTCTAGAGTGGGCTTCCCACCCTTAAAGCGTAAAGTTTATGTTAGTTGTCCAGGAGTGCCGTAG
TCCTGTCGGAAGCTTTAGTGTGAGCCTCTCACGAATAAGCTCGAGATTAGACTCCGTTTGCAAGCCTAAAAAAAA
AAAAAAAAAAAAAAAAAAAAAAAAAAAAAAAAAAAAAAAAAA

>ZYMVΔ (deletion from positions 8551 to 8640 of ZYMV-wt)

>ZYMVΔ-αGFP (insert between positions 8550 and 8641 of ZYMV-wt),
GGTGCGCCGGTGCCGTATCCGGATCCGCTGGAACCGgcccagccggccATGGCTCAGGTGCAGCTGGTGGAGTCT
GGGGGAGCCTTGGTGCAGCCGGGGGGGTCTCTGAGACTCTCCTGTGCAGCCTCTGGATTCCCCGTCAATCGCTAT
AGTATGAGGTGGTACCGCCAGGCTCCAGGGAAGGAGCGCGAGTGGGTCGCGGGTATGAGTAGTGCTGGTGATCGT

TCAAGTTATGAAGACTCCGTGAAGGGCCGATTCACCATCTCCAGAGACGACGCCAGGAATACGGTGTATCTGCAA
ATGAACAGCCTGAAACCTGAGGACACGGCCGTGTATTACTGTAATGTCAATGTGGGCTTTGAGTACTGGGGCCAG
GGGACCCAGGTCACCGTCTCCTCAgcggccgcc**GAACAAAAACTCATCTCAGAAGAGGAT**gcagctgca

αGFP nanobody in green, **E** and **c-Myc** epitopes on yellow and blue background, respectively, and spacers in blue.

>ZYMVΔ-αGFP-F2A (insert between positions 8550 and 8641 of ZYMV-wt)
GGTGCGCCGGTGCCGTATCCGGATCCGCTGGAACCGgcccagccggcc**ATGGCTCAGGTGCAGCTGGTGGAGTCT**
GGGGGAGCCTTGGTGCAGCCGGGGGGGTCTCTGAGACTCTCCTGTGCAGCCTCTGGATTCCCCGTCAATCGCTAT
AGTATGAGGTGGTACCGCCAGGCTCCAGGGAAGGAGCGCGAGTGGGTCGCGGGTATGAGTAGTGCTGGTGATCGT
TCAAGTTATGAAGACTCCGTGAAGGGCCGATTCACCATCTCCAGAGACGACGCCAGGAATACGGTGTATCTGCAA
ATGAACAGCCTGAAACCTGAGGACACGGCCGTGTATTACTGTAATGTCAATGTGGGCTTTGAGTACTGGGGCCAG
GGGACCCAGGTCACCGTCTCCTCAgcggccgcc**GAACAAAAACTCATCTCAGAAGAGGAT**gcagctgca**GGAAGC**
GGAGTGAAACAGACTTTGAATTTTGACCTTCTCAAGTTGGCGGGAGACGTGGAGTCCAACCCTGGACCT

αGFP nanobody in green, **F2A** peptide in blue (splicing position underlined), **E** and **c-Myc** epitopes on yellow and blue background, respectively, and spacers in blue.

Figure S2. Nucleotide sequences of TEV-wt (GenBank accession number DQ986288, including silent mutations **G273A** and A1119G in red) and the derived recombinant viruses TEV-αGFP, and TEV-αGFP-F2A. The boundaries of viral cistrons are indicated on blue background. **Start** and **stop** codons are underlined. Heterologous sequences were inserted between nucleotide positions **8517** and **8518** (underlined). cDNAs corresponding to the anti-green fluorescent protein (**αGFP**) nanobody, and pircornavirus **F2A** peptides are in green and blue, respectively.

```
>TEV-wt (DQ986288, G273A, A1119G); cistrons boundaries are indicated on blue
background; start and stop codons are underlined
AAAATAACAAATCTCAACACAACATATACAAAACAAACGAATCTCAAGCAATCAAGCATTCTACTTCTATTGCAG
CAATTTAAATCATTTCTTTTAAAGCAAAAGCAATTTTCTGAAAATTTTCACCATTTACGAACGATAGCCATGGCA
CTCATCTTTGGCACAGTCAACGCTAACATCCTGAAGGAAGTGTTCGGTGGAGCTCGTATGGCTTGCGTTACCAGC
GCACATATGGCTGGAGCGAATGGAAGCATTTTGAAGAAGGCAGAAGAAACCTCTCGTGCAATCATGCACAAACCA
GTGATCTTCGGAGAAGACTACATTACCGAGGCAGACTTGCCTTACACACCACTCCATTTAGAGGTCGATGCTGAA
ATGGAGCGGATGTATTATCTTGGTCGTCGCGCGCTCACCCATGGCAAGAGACGCAAAGTTTCTGTGAATAACAAG
AGGAACAGGAGAAGGAAAGTGGCCAAAACGTACGTGGGGCGTGATTCCATTGTTGAGAAGATTGTAGTGCCCCAC
ACCGAGAGAAAGGTTGATACCACAGCAGCAGTGGAAGACATTTGCAATGAAGCTACCACTCAACTTGTGCATAAT
AGTATGCCAAAGCGTAAGAAGCAGAAAAACTTCTTGCCCGCCACTTCACTAAGTAACGTGTATGCCCAAACTTGG
AGCATAGTGCGCAAACGCCATATGCAGGTGGAGATCATTAGCAAGAAGAGCGTCCGAGCGAGGGTCAAGAGATTT
GAGGGCTCGGTGCAATTGTTCGCAAGTGTGCGTCACATGTATGGCGAGAGGAAAAGGGTGGACTTACGTATTGAC
AACTGGCAGCAAGAGACACTTCTAGACCTTGCTAAAAGATTTAAGAATGAGAGAGTGGATCAATCGAAGCTCACT
TTTGGTTCAAGTGGCCTAGTTTTGAGGCAAGGCTCGTACGGACCTGCGCATTGGTATCGACATGGTATGTTCATT
GTACGCGGTCGGTCGGATGGGATGTTGGTGGATGCTCGTGCGAAGGTAACGTTCGCTGTTTGTCACTCAATGACA
CATTATAGCGACAAATCAATCTCTGAGGCATTCTTCATACCATACTCTAAGAAATTCTTGGAGTTGAGGCCAGAT
GGAATCTCCCATGAGTGTACAAGAGGAGTATCAGTTGAGCGGTGCGGTGAGGTGGCTGCAATCCTGACACAAGCA
CTTTCACCGTGTGGTAAGATCACATGCAAACGTTGCATGGTTGAAACACCTGACATTGTTGAGGGTGAGTCGGGA
GACAGTGTCACCAACCAAGGTAAGCTCCTAGCAATGCTGAAAGAACAGTATCCAGATTTCCCAATGGCCGAGAAA
CTACTCACAAGGTTTTTGCAACAGAAATCACTAGTAAATACAAATTTGACAGCCTGCGTGAGCGTCAAACAACTC
ATTGGTGACCGCAAACAAGCTCCATTCACACACGTACTGGCTGTCAGCGAAATTCTGTTTAAAGGCAATAAACTA
ACAGGGGCCGATCTCGAAGAGGCAAGCACACATATGCTTGAAATAGCAAGGTTCTTGAACAATCGCACTGAAAAT
ATGCGCATTGGCCACCTTGGTTCTTTCAGAAATAAAATCTCATCGAAGGCCCATGTGAATAACGCACTCATGTGT
GATAATCAACTTGATCAGAATGGGAATTTTATTTGGGGACTAAGGGGTGCACACGCAAAGAGGTTTCTTAAAGGA
TTTTTCACTGAGATTGACCCAAATGAAGGATACGATAAGTATGTTATCAGGAAACATATCAGGGGTAGCAGAAAG
CTAGCAATTGGCAATTTGATAATGTCAACTGACTTCCAGACGCTCAGGCAACAAATTCAAGGCGAAACTATTGAG
CGTAAAGAAATTGGGAATCACTGCATTTCAATGCGGAATGGTAATTACGTGTACCCATGTTGTTGTGTTACTCTT
GAAGATGGTAAGGCTCAATATTCGGATCTAAAGCATCCAACGAAGAGACATCTGGTCATTGGCAACTCTGGCGAT
TCAAAGTACCTAGACCTTCCAGTTCTCAATGAAGAGAAAATGTATATAGCTAATGAAGGTTATTGCTACATGAAC
ATTTTCTTTGCTCTACTAGTGAATGTCAAGGAAGAGGATGCAAAGGACTTCACCAAGTTTATAAGGGACACAATT
GTTCCAAAGCTTGGAGCGTGGCCAACAATGCAAGATGTTGCAACTGCATGCTACTTACTTTCCATTCTTTACCCA
GATGTCCTGAGTGCTGAATTACCCAGAATTTTGGTTGATCATGACAACAAAACAATGCATGTTTTGGATTCGTAT
GGGTCTAGAACGACAGGATACCACATGTTGAAAATGAACACAACATCCCAGCTAATTGAATTCGTTCATTCAGGT
TTGGAATCCGAAATGAAAACTTACAATGTTGGAGGGATGAACCGAGATATGGTCACACAAGGTGCAATTGAGATG
TTGATCAAGTCCATATACAAACCACATCTCATGAAGCAGTTACTTGAGGAGGAGCCATACATAATTGTCCTGGCA
ATAGTCTCCCCTTCAATTTTAATTGCCATGTACAACTCTGGAACTTTTGAGCAGGCGTTACAAATGTGGTTGCCA
AATACAATGAGGTTAGCTAACCTCGCTGCCATCTTGTCAGCCTTGGCGCAAAAGTTAACTTTGGCAGACTTGTTC
GTCCAGCAGCGTAATTTGATTAATGAGTATGCGCAGGTAATTTTGGACAATCTGATTGACGGTGTCAGGGTTAAC
CATTCGCTATCCCTAGCAATGGAAATTGTTACTATTAAGCTGGCCACCCAAGAGATGGACATGGCGTTGAGGGAA
GGTGGCTATGCTGTGACCTCTGAAAAGGTGCATGAAATGTTGGAAAAAAACTATGTAAAGGCTTTGAAGGATGCA
TGGGACGAATTAACTTGGTTGGAAAAATTCTCCGCAATCAGGCATTCAAGAAAGCTCTTGAAATTTGGGCGAAAG
CCTTTAATCATGAAAAACACCGTAGATTGCGGCGGACATATAGACTTGTCTGTGAAATCGCTTTTCAAGTTCCAC
TTGGAACTCCTGAAGGGAACCATCTCAAGAGCCGTAAATGGTGGTGCAAGAAAGGTAAGAGTAGCGAAGAATGCC
```

ATGACAAAAGGGGTTTTTCTCAAAATCTACAGCATGCTTCCTGACGTCTACAAGTTTATCACAGTCTCGAGTGTC
CTTTCCTTGTTGTTGACATTCTTATTTCAAATTGACTGCATGATAAGGGCACACCGAGAGGCGAAGGTTGCTGCA
CAGTTGCAGAAAGAGAGCGAGTGGGACAATATCATCAATAGAACTTTCCAGTATTCTAAGCTTGAAAATCCTATT
GGCTATCGCTCTACAGCGGAGGAAAGACTCCAATCAGAACACCCCGAGGCTTTCGAGTACTACAAGTTTTGCATT
GGAAAGGAAGACCTCGTTGAACAGGCAAAACAACCGGAGATAGCATACTTTGAAAAGATTATAGCTTTCATCACA
CTTGTATTAATGGCTTTTGACGCTGAGCGGAGTGATGGAGTGTTCAAGATACTCAATAAGTTCAAAGGAATACTG
AGCTCAACGGAGAGGGAGATCATCTACACGCAGAGTTTGGATGATTACGTTACAACCTTTGATGACAATATGACA
ATCAACCTCGAGTTGAATATGGATGAACTCCACAAGACGAGCCTTCCTGGAGTCACTTTTAAGCAATGGTGGAAC
AACCAAATCAGCCGAGGCAACGTGAAGCCACATTATAGAACTGAGGGGCACTTCATGGAGTTTACCAGAGATACT
GCGGCATCGGTTGCCAGCGAGATATCACACTCACCCGCAAGAGATTTTCTTGTGAGAGGTGCTGTTGGATCTGGA
AAATCCACAGGACTTCCATACCATTTATCAAAGAGAGGGAGAGTGTTAATGCTTGAGCCTACCAGACCACTCACA
GATAACGTGCACAAGCAACTGAGAAGTGAACCATTTAACTGCTTCCCAACTTTGAGGATGAGAGGGAAGTCAACT
TTTGGGTCATCACCGATTACAGTCATGACTAGTGGATTCGCTTTACACCATTTTGCACGAAACATAGCTGAGGTA
AAAACATACGATTTTGTCATAATTGATGAATGTCATGTGAATGATGCTTCTGCTATAGCGTTTAGGAATCTACTG
TTTGAACATGAATTTGAAGGAAAAGTCCTCAAAGTGTCAGCCACACCACCAGGTAGAGAAGTTGAATTCACAACT
CAGTTTCCCGTGAAACTCAAGATAGAAGAGGCTCTTAGCTTTCAGGAATTTGTAAGTTTACAAGGGACAGGTGCC
AACGCCGATGTGATTAGTTGTGGCGACAACATACTAGTATATGTTGCTAGCTACAATGATGTTGATAGTCTTGGC
AAGCTCCTTGTGCAAAAGGGATACAAAGTGTCGAAGATTGATGGAAGAACAATGAAGAGTGGAGGAACTGAAATA
ATCACTGAAGGTACTTCAGTGAAAAAGCATTTCATAGTCGCAACTAATATTATTGAGAATGGTGTAACCATTGAC
ATTGATGTAGTTGTGGATTTTGGGACTAAGGTTGTACCAGTTTTGGATGTGGACAATAGAGCGGTGCAGTACAAC
AAAACTGTGGTGAGTTATGGGGAGCGCATCCAAAGACTCGGTAGAGTTGGGCGACACAAGGAAGGAGTAGCACTT
CGAATTGGCCAAACAAATAAAACACTGGTTGAAATTCCAGAAATGGTTGCCACTGAAGCTGCCTTTCTATGCTTC
ATGTACAATTTGCCAGTGACAACACAGAGTGTTTCAACCACACTGCTGGAAAATGCCACATTATTACAAGCTAGA
ACTATGGCACAGTTTGAGCTATCATATTTTTACACAATTAATTTTGTGCGATTTGATGGTAGTATGCATCCAGTC
ATACATGACAAGCTGAAGCGCTTTAAGCTACACACTTGTGAGACATTCCTCAATAAGTTGGCGATCCCAAATAAA
GGCTTATCCTCTTGGCTTACGAGTGGAGAGTATAAGCGACTTGGTTACATAGCAGAGGATGCTGGCATAAGAATC
CCATTCGTGTGCAAAGAAATTCCAGACTCCTTGCATGAGGAAATTTGGCACATTGTAGTCGCCCATAAAGGTGAC
TCGGGTATTGGGAGGCTCACTAGCGTACAGGCAGCAAAGGTTGTTTATACTCTGCAAACGGATGTGCACTCAATT
GCGAGGACTCTAGCATGCATCAATAGACTCATAGCACATGAACAAATGAAGCAGAGTCATTTTGAAGCCGCAACT
GGGAGAGCATTTTCCTTCACAAATTACTCAATACAAAGCATATTTGACACGCTGAAAGCAAATTATGCTACAAAG
CATACGAAAGAAAATATTGCAGTGCTTCAGCAGGCAAAAGATCAATTGCTAGAGTTTTCGAACCTAGCAAAGGAT
CAAGATGTCACGGGTATCATCCAAGACTTCAATCACCTGGAAACTATCTATCTCCAATCAGATAGCGAAGTGGCT
AAGCATCTGAAGCTTAAAAGTCACTGGAATAAAAGCCAAATCACTAGGGACATCATAATAGCTTTGTCTGTGTTA
ATTGGTGGTGGATGGATGCTTGCAACGTACTTCAAGGACAAGTTCAATGAACCAGTCTATTTCCAAGGGAAGAAG
AATCAGAAGCACAAGCTTAAGATGAGAGAGGCGCGTGGGGCTAGAGGGCAATATGAGGTTGCAGCGGAGCCAGAG
GCGCTAGAACATTACTTTGGAAGCGCATATAATAACAAAGGAAAGCGCAAGGGCACCACGAGAGGAATGGGTGCA
AAGTCTCGGAAATTCATAAACATGTATGGGTTTGATCCAACTGATTTTTCATACATTAGGTTTGTGGATCCATTG
ACAGGTCACACTATTGATGAGTCCACAAACGCACCTATTGATTTAGTGCAGCATGAGTTTGGAAAGGTTAGAACA
CGCATGTTAATTGACGATGAGATAGAGCCTCAAAGTCTTAGCACCCACACCACAATCCATGCTTATTTGGTGAAT
AGTGGCACGAAGAAAGTTCTTAAGGTTGATTTAACACCACACTCGTCGCTACGTGCGAGTGAGAAATCAACAGCA
ATAATGGGATTTCCTGAAAGGGAGAATGAATTGCGTCAAACCGGCATGGCAGTGCCAGTGGCTTATGATCAATTG
CCACCAAAGAGTGAGGACTTGACGTTTGAAGGAGAAAGCTTGTTTAAGGGACCACGTGATTACAACCCGATATCG
AGCACCATTTGTCACTTGACGAATGAATCTGATGGGCACACAACATCGTTGTATGGTATTGGATTTGGTCCCTTC
ATCATTACAAACAAGCACTTGTTTAGAAGAAATAATGGAACACTGTTGGTCCAATCACTACATGGTGTATTCAAG
GTCAAGAACACCACGACTTTGCAACAACACCTCATTGATGGGAGGGACATGATAATTATTCGCATGCCTAAGGAT
TTCCCACCATTTCCTCAAAAGCTGAAATTTAGAGAGCCACAAAGGGAAGAGCGCATATGTCTTGTGACAACCAAC
TTCCAAACTAAGAGCATGTCTAGCATGGTGTCAGACACTAGTTGCACATTCCCTTCATCTGATGGCATATTCTGG
AAGCATTGGATTCAAACCAAGGATGGGCAGTGTGGCAGTCCATTAGTATCAACTAGAGATGGGTTCATTGTTGGT
ATACACTCAGCATCGAATTTCACCAACACAAACAATTATTTCACAAGCGTGCCGAAAAACTTCATGGAATTGTTG
ACAAATCAGGAGGCGCAGCAGTGGGTTAGTGGTTGGCGATTAAATGCTGACTCAGTATTGTGGGGGGGCCATAAA
GTTTTCATGAGCAAACCTGAAGAGCCTTTTCAGCCAGTTAAGGAAGCGACTCAACTCATGAGTGAATTGGTGTAC
TCGCAAGGGGAGAAGAGGAAATGGGTCGTGGAAGCACTGTCAGGGAACTTGAGGCCAGTGGCTGAGTGTCCCAGT
CAGTTAGTCACAAAGCATGTGGTTAAAGGAAAGTGTCCCCTCTTTGAGCTCTACTTGCAGTTGAATCCAGAAAAG

GAAGCATATTTTAAACCGATGATGGGAGCATATAAGCCAAGTCGACTTAATAGAGAGGCGTTCCTCAAGGACATT
CTAAAATATGCTAGTGAAATTGAGATTGGGAATGTGGATTGTGACTTGCTGGAGCTTGCAATAAGCATGCTCATC
ACAAAGCTCAAGGCGTTAGGATTCCCAACTGTGAACTACATCACTGACCCAGAGGAAATTTTTAGTGCATTGAAT
ATGAAAGCAGCTATGGGAGCACTATACAAAGGCAAGAAGAAAGAAGCTCTCAGCGAGCTCACACTAGATGAGCAG
GAGGCAATGCTCAAAGCAAGTTGCCTGCGACTGTATACGGGAAAGCTGGGAATTTGGAATGGCTCATTGAAAGCA
GAGTTGCGTCCAATTGAGAAGGTTGAAAACAACAAAACGCGAACTTTCACAGCAGCACCAATAGACACTCTTCTT
GCTGGTAAAGTTTGCGTGGATGATTTCAACAATCAATTTTATGATCTCAACATAAAGGCACCATGGACAGTTGGT
ATGACTAAGTTTTATCAGGGGTGGAATGAATTGATGGAGGCTTTACCAAGTGGGTGGGTGTATTGTGACGCTGAT
GGTTCGCAATTCGACAGTTCCTTGACTCCATTCCTCATTAATGCTGTATTGAAAGTGCGACTTGCCTTCATGGAG
GAATGGGATATTGGTGAGCAAATGCTGCGAAATTTGTACACTGAGATAGTGTATACACCAATCCTCACACCGGAT
GGTACTATCATTAAGAAGCATAAAGGCAACAATAGCGGGCAACCTTCAACAGTGGTGGACAACACACTCATGGTC
ATTATTGCAATGTTATACACATGTGAGAAGTGTGGAATCAACAAGGAAGAGATTGTGTATTACGTCAATGGCGAT
GACCTATTGATTGCCATTCACCCAGATAAAGCTGAGAGGTTGAGTGGATTCAAAGAATCTTTCGGAGAGTTGGGC
CTGAAATATGAATTTGACTGCACCACCAGGGACAAGACACAGTTGTGGTTCATGTCACACAGGGCTTTGGAGAGG
GATGGCATGTATATACCAAAGCTAGAAGAAGAAAGGATTGTTTCTATTTTGGAATGGGACAGATCCAAAGAGCCG
TCACATAGGCTTGAAGCCATCTGTGCATCAATGATCGAAGCATGGGGTTATGACAAGCTGGTTGAAGAAATCCGC
AATTTCTATGCATGGGTTTTGGAACAAGCGCCGTATTCACAGCTTGCAGAAGAAGGAAAGGCGCCATATCTGGCT
GAGACTGCGCTTAAGTTTTTGTACACATCTCAGCACGGAACAAACTCTGAGATAGAAGAGTATTTAAAAGTGTTG
TATGATTACGATATTCCAACGACTGAGAATCTTTATTTTCAGAGTGGCACTGTGGGTGCTGGTGTTGACGCTGGT
AAGAAGAAAGATCAAAAGGATGATAAAGTCGCTGAGCAGGCTTCAAAGGATAGGGATGTTAATGCTGGAACTTCA
GGAACATTCTCAGTTCCACGAATAAATGCTATGGCCACAAAACTTCAATATCCAAGGATGAGGGGAGAGGTGGTT
GTAAACTTGAATCACCTTTTAGGATACAAGCCACAGCAAATTGATTTGTCAAATGCTCGAGCCACACATGAGCAG
TTTGCCGCGTGGCATCAGGCAGTGATGACAGCCTATGGAGTGAATGAAGAGCAAATGAAAATATTGCTAAATGGA
TTTATGGTGTGGTGCATAGAAAATGGGACTTCCCCAAATTTGAACGGAACTTGGGTTATGATGGATGGTGAGGAG
CAAGTTTCATACCCGCTGAAACCAATGGTTGAAAACGCGCAGCCAACACTGAGGCAAATTATGACACACTTCAGT
GACCTGGCTGAAGCGTATATTGAGATGAGGAATAGGGAGCGACCATACATGCCTAGGTATGGTCTACAGAGAAAC
ATTACAGACATGAGTTTGTCACGCTATGCGTTCGACTTCTATGAGCTAACTTCAAAAACACCTGTTAGAGCGAGG
GAGGCGCATATGCAAATGAAAGCTGCTGCAGTACGAAACAGTGGAACTAGGTTATTTGGTCTTGATGGCAACGTG
GGTACTGCAGAGGAAGACACTGAACGGCACACAGCGCACGATGTGAACCGTAACATGCACACACTATTAGGGGTC
CGCCAGTGATAGTTTCTGCGTGTCTTTGCTTTCCGCTTTTAAGCTTATTGTAATATATATGAATAGCTATTCACA
GTGGGACTTGGTCTTGTGTTGAATGGTATCTTATATGTTTTAATATGTCTTATTAGTCTCATTACTTAGGCGAAC
GACAAAGTGAGGTCACCTCGGTCTAATTCTCCTATGTAGTGCGAGAAAAAAAAAAAAAAAAAAAAAAAAAAAAAA
AAAAAAAAAAAAAAA

>TEV-αGFP (insert between possitions 8517 and 8518 of TEV-wt)
TCAGGTACAGGTGCGCCGGTGCCGTATCCGGATCCGCTGGAACCGgcccagccggccATGGCTCAGGTGCAGCTG
GTGGAGTCTGGGGGAGCCTTGGTGCAGCCGGGGGGGTCTCTGAGACTCTCCTGTGCAGCCTCTGGATTCCCCGTC
AATCGCTATAGTATGAGGTGGTACCGCCAGGCTCCAGGGAAGGAGCGCGAGTGGGTCGCGGGTATGAGTAGTGCT
GGTGATCGTTCAAGTTATGAAGACTCCGTGAAGGGCCGATTCACCATCTCCAGAGACGACGCCAGGAATACGGTG
TATCTGCAAATGAACAGCCTGAAACCTGAGGACACGGCCGTGTATTACTGTAATGTCAATGTGGGCTTTGAGTAC
TGGGGCCAGGGGACCCAGGTCACCGTCTCCTCAgcggccgccGAACAAAAACTCATCTCAGAAGAGGATgcagct
gca

Duplicated three initial codons (silent mutations) of TEV CP are in black. **αGFP** nanobody in green, **E** and **c-Myc** epitopes on yellow and blue background, respectively, and spacers in blue.

>TEV-αGFP-F2A (insert between possitions 8517 and 8518 of TEV-wt)
TCAGGTACAGGTGCGCCGGTGCCGTATCCGGATCCGCTGGAACCGgcccagccggccATGGCTCAGGTGCAGCTG
GTGGAGTCTGGGGGAGCCTTGGTGCAGCCGGGGGGGTCTCTGAGACTCTCCTGTGCAGCCTCTGGATTCCCCGTC
AATCGCTATAGTATGAGGTGGTACCGCCAGGCTCCAGGGAAGGAGCGCGAGTGGGTCGCGGGTATGAGTAGTGCT
GGTGATCGTTCAAGTTATGAAGACTCCGTGAAGGGCCGATTCACCATCTCCAGAGACGACGCCAGGAATACGGTG
TATCTGCAAATGAACAGCCTGAAACCTGAGGACACGGCCGTGTATTACTGTAATGTCAATGTGGGCTTTGAGTAC
TGGGGCCAGGGGACCCAGGTCACCGTCTCCTCAgcggccgccGAACAAAAACTCATCTCAGAAGAGGATgcagct

gca**GGAAGCGGAGTGAAACAGACTTTGAATTTTGACCTTCTCAAGTTGGCGGGAGACGTGGAGTCCAACCCTGGA**
CCT

Duplicated three initial codons (silent mutations) of TEV CP are in black. **αGFP** nanobody in green, **F2A** peptide in blue (splicing position underlined), **E** and **c-Myc** epitopes on yellow and blue background, respectively, and spacers in blue.

Figure S3. Nucleotide sequence of the recombinant enhanced green fluorescent protein (eGFP) with the **Twin-Strep tag** (in red, spacers in grey) produced in *Escherichia coli*. **Start** and **stop** codons are underlined.

ATGGTGAGCAAGGGCGAGGAGCTGTTCACCGGGGTGGTGCCCATCCTGGTCGAGCTGGACGGCGACGTAAACGGC
CACAAGTTCAGCGTGTCCGGCGAGGGCGAGGGCGATGCCACCTACGGCAAGCTGACCCTGAAGTTCATCTGCACC
ACCGGCAAGCTGCCCGTGCCCTGGCCCACCCTCGTGACCACCCTGACCTACGGCGTGCAGTGCTTCAGCCGCTAC
CCCGACCACATGAAGCAGCACGACTTCTTCAAGTCCGCCATGCCCGAAGGCTACGTCCAGGAGCGCACCATCTTC
TTCAAGGACGACGGCAACTACAAGACCCGCGCCGAGGTGAAGTTCGAGGGCGACACCCTGGTGAACCGCATCGAG
CTGAAGGGCATCGACTTCAAGGAGGACGGCAACATCCTGGGGCACAAGCTGGAGTACAACTACAACAGCCACAAC
GTCTATATCATGGCCGACAAGCAGAAGAACGGCATCAAGGTGAACTTCAAGATCCGCCACAACATCGAGGACGGC
AGCGTGCAGCTCGCCGACCACTACCAGCAGAACACCCCCATCGGCGACGGCCCCGTGCTGCTGCCCGACAACCAC
TACCTGAGCACCCAGTCCGCCCTGAGCAAAGACCCCAACGAGAAGCGCGATCACATGGTCCTGCTGGAGTTCGTG
ACCGCCGCCGGGATCACTCTCGGCATGGACGAGCTGTACAAGAGCGCA**TGGAGTCATCCTCAATTCGAGAAA**GGT
GGAGGTTCTGGCGGTGGATCGGGAGGTTCAGCG**TGGAGCCACCCGCAGTTCGAAAAA**TCCGGA**TGA**
